# Edible bird’s nest, an Asian health food supplement, possesses anti-inflammatory responses in restoring the symptoms of atopic dermatitis: An analysis of signaling cascades

**DOI:** 10.3389/fphar.2022.941413

**Published:** 2022-09-20

**Authors:** Queenie Wing Sze Lai, Qunyan Fan, Brody Zhongyu Zheng, Yanxian Chen, Tina Tingxia Dong, Karl Wah Keung Tsim

**Affiliations:** ^1^ Shenzhen Research Institute, The Hong Kong University of Science and Technology, Shenzhen, China; ^2^ Division of Life Science and Center for Chinese Medicine R&D, The Hong Kong University of Science and Technology, Kowloon, Hong Kong, Hong Kong SAR, China; ^3^ Bird’s Nest Research Institute of Yan Palace, Xiamen Yan Palace Seelong Food Co., Ltd., Xiamen, Fujian, China

**Keywords:** bird’s nest, sialic acid, TNF-α signaling, reactive oxygen species, filaggrin, skin dermatitis

## Abstract

Edible bird’s nest (EBN) is a Chinese delicacy possessing skin rejuvenating functions. To verify skin anti-inflammatory function of EBN, water extract and enzymatic digest of EBN, as well as the major sialic acid, N-acetyl neuraminic acid (NANA), were probed in TNF-α-treated HaCaT keratinocytes. The mRNA expressions of pro-inflammatory cytokines, e.g., IL-1β, IL-6, TNF-α, and an enzyme responsible for inflammatory response, i.e., Cox-2, as well as filaggrin and filaggrin-2, were markedly altered after treating with different preparations of EBN. The EBN-mediated responses could be accounted by its robust reduction of reactive oxygen species (ROS), NF-κB signaling and phosphorylation of p38 MAPK and JNK, as triggered by TNF-α-induced inflammation. The anti-inflammatory response of EBN was further supported in animal model. In 2,4-dinitrochlorobenzene (DNCB)-induced dermatitic mice, the effects on skin thickness, severity level of damage and scratching behavior, exerted by DNCB, were reversed after EBN treatments, in dose-dependent manners. In parallel, the levels of immune cells and pro-inflammatory cytokines in dermatitic skin were markedly reduced by treatment of EBN preparations. In general, NANA and enzymatic digest of EBN showed better anti-inflammatory responses in both models of *in vitro* and *in vivo*. These lines of evidence therefore suggest the possible application of EBN in treating atopic dermatitis.

## Introduction

Edible bird’s nests (EBNs; Yan Wo) are solidified saliva secreted by *Aerodramus fuciphagus* and *Aerodramus maximus*, which are being consumed in Asia for centuries. Today, EBNs in forms of cup or stripe are produced in Southeast Asian countries, e.g., Indonesia, Malaysia, Vietnam, and Thailand. EBN has been valued greatly by Chinese for its functions in nourishing “*Yin*” and strengthening “*Lung*”, i.e., in regulating immunity and skin condition (Li et al., 2021): these functions are being supported by modern pharmacological evaluation. EBN has been known to strengthen respiratory system and to promote skin function. Besides, the intake of EBN leads to enhancement of immunity, alleviation of asthma, and improvement of skin complexion (Chye et al., 2017), anti-aging and promotion of wound-healing (Hwang et al., 2020), restoration of skin lightness (Chan et al., 2015), and facilitation of skin moisturization (Lai et al., 2021). EBN contains a rich source of proteins, providing glycoproteins and 16 types of essential amino acids (Halimi et al., 2015). The protein content in EBN is over 50% by weight, existing in macromolecule(s) that is not pharmacological useful as a skin-care product, due to limited bioavailability or skin penetration (Chan et al., 2015; Lai et al., 2021). Indeed, methods have been developed to maximize the extracting efficiency of protein/peptide, as well as the specific form of sialic acid, N-acetyl neuraminic acid (NANA), from EBN (Wong et al., 2018a).

Atopic dermatitis (AD), also known as eczema, is a common chronic inflammatory skin disease, occurring at all ages due to genetic and/or environmental factors. Patients suffering from AD are having dry, itchy, and scaly skin with rashes and cutaneous infections. AD affects up to 20% children and 10% adults in developed countries, and about 230 million people are affected by AD in 2010 (Langan et al., 2020). The loss-of-function mutation of *filaggrin* gene is the major genetic cause of AD (De Jongh et al., 2008), and in parallel the diminished expression of filaggrin-2 is leading to the psoriatic lesion (Kypriotou et al., 2012). In external causes, the exposure to chemicals, or having emotional stress, could cause the skin to develop AD symptoms. Besides, those people living in cities and dry climates are frequently affected. Today, no perfect cure is found for AD.

Skin inflammation is the vascular and cellular responses to resolve insult by irritants and to restore normal skin homeostasis (Mitchell, 1997). The cellular inflammatory responses trigger the recruitment of immune cells to rescue the damages of keratinocytes and macrophages in skin epidermis. The recruitment of immune cells to the site of injury is mediated by cytokines and chemokines, and which thereafter triggers various signaling pathways during the inflammation, e.g., NF-κB (Wullaert et al., 2011), MAPK (Wang et al., 2009), mTOR (Cibrian et al., 2020), JAK-STAT (Bao et al., 2013), Wnt (Augustin, 2015), and Notch (Dumortier et al., 2010). Thus, the activations of these signaling pathways are reported to induce skin inflammation.

In cultured keratinocytes, the treatment of EBN extract could lead to the suppression of TNF-α/IFN-γ-induced expression of chemokines (Hwang et al., 2020). In parallel, the enzymatic digest of EBN was shown to robustly induce the expression of filaggrin and filaggrin-2 in cultured keratinocytes. Besides, NANA is a negatively charged sugar, which facilitates the Ca^2+^ binding to proteins and increases the cellular viscosity (Hofbauer et al., 1998). Moreover, NANA can modulate the glycoconjugate structure via non-enzymatic sialylation, suggesting its ability to block those enzymes involving in allergic reactions (Kai et al., 1990). In parallel, sialic acid was demonstrated to inhibit adherence, chemotaxis, and spontaneous motility of neutrophils (Görög and Kovács, 1978). Herein, we hypothesize that EBN can suppress the production of pro-inflammatory cytokines during inflammation. Both *in vitro* and *in vivo* models were established here to illustrate the role of EBN in anti-inflammatory responses. Besides, the application of EBN-containing cream could be further developed for AD treatment.

## Materials and methods

### Preparation of EBN extract and digestion

White EBN, originated from Malaysia house production site in standard “cup” grade, was purchased in Hong Kong market. The EBN sample was stored at room temperature. The raw EBN was weighed and soaked in 1:100 (w/v) double deionized (DDI) water for overnight. On the next day, the soaked EBN was rinsed with DDI water 3 times to remove inorganic materials, and then which was boiled at 98 ± 2 °C with 1 : 30 (w/v) DDI water for 8 h under constant stirring. The stewed EBN was filtered, and the filtrate was collected for lyophilization. The lyophilized powder was considered as EBN extract. To prepare the digested EBN, the dried EBN extract was weighed and digested with 1 : 100 (w/v) simulated gastric fluid (SGF; without enzyme; containing 0.07 M hydrochloric acid and 0.1 M sodium chloride) and 7.6% (w/w) pepsin (from porcine gastric mucosa lyophilized powder, ≥ 2,500 units/mg protein) (Sigma-Aldrich, St Louis, MO) for 48 h at 37°C for complete digestion, as described before (Lai et al., 2021). After 48 h, the enzymatic digestion was terminated by neutralizing by 0.7 M sodium hydroxide solution. Lyophilization was performed to prepare the EBN digest for subsequent experiments.

### HPLC analysis

HPLC-UV chromatographic separation of EBN digest was performed on an Agilent HPLC 1200 series system (Agilent, Waldbronn, Germany) equipped with a diode array detector. The EBN digest was reconstituted in DDI water and re-separated by Superdex 75 gel filtration column (GE Healthcare, Little Chalfont, United Kingdom). The UV absorbance was measured at 214 nm (peptide) and 280 nm (protein), as described (Lai et al., 2021). For the peptide fingerprint, EBN samples were separated by a Symmetry Sheild™ RP C18 column (5 mm i.d., 250 mm × 4.6 mm). The mobile phase was composed of 0.1% trifluoracetic acid in water (A) and acetonitrile (B) using a gradient program: 0–10 min, linear gradient 5–15% (B); 10–30 min, linear gradient 15–30% (B); 30–50 min, isocratic gradient 30% (B); 50–60 min, linear gradient 30–5% (B); a pre-equilibration period of 20 min was used between each run. The flow rate was 0.6 mL/min; the column temperature was 25°C; and the injection volume was 10 μL. The DAD wavelength was set to 214 nm (peptide) and 280 nm (protein), as described (Wong et al., 2017).

### LC-MS/MS analysis

N-Acetyl-d-neuraminic acid (NANA) was purchased from Sigma-Aldrich, as standard stock solution (Std-stock). The liquid chromatograph was equipped with an Agilent 6,410 Triple Quad MS/MS and an Eclipse XDB-C18 column (2.1 × 100 mm, 3.5 µm particle size). The injection volume was 2 µL. Five-min linear gradient at flow rates of 0.4 mL/min between solvent A (Milli-Q water, 0.1% formic acid) and solvent B (acetonitrile, 0.1% formic acid) was used. After reaching 80% B, the system returned to 100% A in 0.5 min. For column equilibration, a total cycle time of 10 min was needed. The retention time of NANA was 0.80–0.85 min. The MS was operated in negative electron spray ionization mode. A capillary voltage of 3.5 kV and a cone voltage of 10 V were applied. The source temperature was 100°C, and the desolvation temperature was 325°C. Ultra-high purity nitrogen was used for cone gas (3.0 L/min), desolvation gas (10.0 L/min) and nebulizing gas (35 psi). For collision induced dissociation (CID), collision energy of 5 eV was used. Negatively single charged ions [M-H]^-^ of NANA (m/z 307.9) were selected as precursor ions for CID. The precursor ion was dissociated into two major product ions (m/z 87.0 and 170.0), and the product ion m/z 87.0 was the most abundant from NANA. An internal standard was applied for normalization. For multiple reaction monitoring, the transitions m/z 307.9 → 87.0 and m/z 307.9 → 170.0 were chosen as the qualifiers, while the transition m/z 307.9 → 87.0 was measured for quantification. For full scanning of total ion chromatograms, the spectra from m/z 50 to m/z 1,000 were recorded. The extracted and digested EBN were diluted, centrifuged (19,000 rcf for 5 min) and filtered before subjected to LC-MS/MS analysis, as described (Wong et al., 2018a).

### Cell culture

Human epidermal keratinocytes, HaCaT cells (AddexBio, San Diego, CA), were cultured in Dulbecco’s modified Eagle’s medium (DMEM), supplemented with 10% (v/v) fetal bovine serum (FBS) and 1% (v/v) penicillin/streptomycin (10,000 U and 10,000 μg/mL) in a humidified atmosphere with 5% CO_2_ at 37°C. All culture reagents were purchased from Thermo Fisher Scientific (Waltham, MA).

### Cell viability test

Cell viability was assessed by MTT assay (Mosmann, 1983). HaCaT keratinocytes were seeded in the 96-well-plate at 1 × 10^5^ cells/mL and incubated for 24 h. After that, cells were treated with EBN water extracts and digested products for 48 h. Then, MTT solution was added to cultures and incubated at 37°C. After the solution was removed, the purple precipitate inside the cells was re-suspended in DMSO. Absorbance was measured at 570 nm in a Multiskan™ FC Microplate Photometer (Thermo Fisher Scientific).

### DNA transfection and luciferase assay

The DNA construct pNF-κB-Luc containing 5 NF-κB response elements, i.e., 5′-GGG AAT TTC CG-3′, and a downstream firefly luciferase-reporter gene. The DNA construct pFLG2-eGFP composes of the vector pEGFP-N1 having a filaggrin-2 promoter, driving the transcription of a red-shifted variant of wild-type green fluorescence protein reporter. Another DNA construct pARE-Luc, containing four antioxidant response elements (ARE: 5′-TGA nnn GCA-3′) and a downstream firefly luciferase-reporter gene, was used. Additionally, the DNA construct pHRE-Luc, containing six hypoxic response elements (HRE: 5′-GTG ACT ACG TGC CTA G-3′) and a downstream firefly luciferase-reporter gene, was adopted. Cultured HaCaT keratinocytes were transfected using jetPRIME reagent (Polyplus Transfection, New York, NY), as described (Lai et al., 2021). In pNF-κB-Luc, pARE-Luc and pHRE-Luc transfected cells, TNF-α at 20 ng/mL was applied for 2 or 6 h with the treatment of NANA, or EBN extract, or EBN digest. Dexamethasone (Sigma-Aldrich) at 10 nM in pNF-κB-Luc assay, or tert-butylhydroquinone (tBHQ) at 20 μM in pARE-Luc and pHRE-Luc assays, was employed as a positive control. Cells were solubilized in lysis buffer, containing 100 mM potassium phosphate buffered saline (PBS, pH 7.8) with 0.2% Triton and 1 mM DTT. After centrifugation (16,000 x g for 5 min at 4°C), 75 µL of cell lysate was transferred to 96-well assay plate and set on GloMax 96 Microplate Luminometer (Promega, Madison, WI). The readings of bioluminescent intensity were then normalized by protein concentration for each lysate. In pFLG2-eGFP transfected cultures, GFP quantification was performed in a FlexStation^®^ three Benchtop Multi-Mode Microplate Reader (Molecular Devices, Sunnyvale, CA). Fluorescence signals were measured under excitation/emission wavelength 488/509 nm. The signals of GFP were normalized to total proteins, determined by Bradford assay (Bio-Rad Laboratories, Hercules, CA).

### Measurement of intracellular ROS generation

HaCaT keratinocytes were seeded on a sterile coverslip (Marienfeld Superior, Lauda-Königshofen, Germany) in 12-well plate at 5 × 10^4^ cells/mL. Cells were stimulated with TNF-α at 5 min with or without 2 h pre-incubation of NANA, EBN extract or EBN digest. N-acetyl-l-cysteine (NAC) at 20 mM pre-incubated for 1 h in cultures was adopted as a positive control. Cultured keratinocytes were stained with 2′-7′ dichlorofluorescin diacetate (DCFH-DA) for 20 min and fixed with 4% paraformaldehyde for 10 min at room temperature. Samples were mounted with ProLong Gold Antifade Mountant with DAPI (Thermo Fisher Scientific), examined by a Leica SP8 Confocal Microscope (Leica Microsystems, Wetzlar, Germany). The fluorescence quantification was performed in a FlexStation^®^ 3 Benchtop Multi-Mode Microplate Reader (Molecular Devices), measured under excitation/emission wavelength 492/527 nm, kinetically for 1 h. The green fluorescence signals were normalized to total protein.

### Total antioxidant capacity

The total antioxidant capacity of EBN was examined by a commercial kit. The reaction mixtures were loaded onto a 96-well plate. Trolox standard curve (0–20 nmol) was prepared according to the instruction manual. One hundred µL sample was prepared with Milli-Q water at concentration indicated. The Cu^2+^ working solution (100 µL) was added. The optical density was measured at 570 nm after 90 min of incubation under dark. The reaction was carried out at room temperature, and the absorption was recorded using a micro-plate spectrophotometer.

### Real-time PCR analysis

The mRNAs encoding IL-1β, IL-6, TNF-α, Cox-2, filaggrin, filaggrin-2, Nrf2, HO-1, HIF-1α, HIF-2α, keratin 1, keratin 10, and glyceraldehyde-3-phosphate dehydrogenase (GAPDH) were quantified by real-time PCR analysis. In cultured HaCaT keratinocytes, total RNA was extracted by using RNAzol^®^ RT RNA isolation reagent (Molecular Research Center, Cincinnati, IO). RNA quality and amount were determined by NanoDrop™ (Thermo Fisher Scientific) at A260/A280 and A260/A230. Total RNA was normalized to 2 μg, and the contaminating genomic DNA was removed with DNase I (New England Biolabs, Hitchin, United Kingdom). Total RNA at 500 ng was reverse transcribed to cDNA using PrimeScript™ RT Reagent Kit (TaKaRa, Kusatsu, Japan). Relative gene quantification was performed by LightCycler^®^ 480 Real-Time PCR System (Roche, Basel, Switzerland). The sequences of specific primers were shown as followed: sense 5′-ATG GCA GAA GTA CCT AAG CTC GC-3′ and antisense 5′-ACA CAA ATT GCA TGG TGA AGT CAG TT-3′ for IL-1β; sense 5′-GAG AGT AGT GAG GAA CAA GCC AGA GC-3′ and antisense 5′-CTA CAT TTG CCG AAG AGC CCT CAG G-3′ for IL-6; sense 5′-ATG AGC ACT GAA AGC ATG ATC CGG-3′ and antisense 5′-GCA ATG ATC CCA AAG TAG ACC TGC CC-3′ for TNF-α; sense 5′-CTG CGA CTC CTT GAC GTT GA-3′ and antisense 5′-GGT CGT GTA GCG GTG AAA GT-3′ for Cox-2; sense 5′-GCT GAA GGA ACT TCT GGA AAA GG-3′ and antisense 5′-GTT GTG GTC TAT ATC CAA GTG ATC-3′ for filaggrin; sense 5′-CTG TGG TCA TTC ATG GAG TGG-3′ and antisense 5′-CCC TAG AAG GGC TAA TGT GTG A-3′ for filaggrin-2; sense 5′-CAC ATC CAG TCA GAA ACC AGT GG-3′ and antisense 5′-GGA ATG TCT GCG CCA AAA GCT G-3′ for Nrf2; sense 5′-CCA GGC AGA GAA TGC TGA GTT C-3′ and antisense 5′-AAG ACT GGG CTC TCC TTG TTG C-3′ for HO-1; sense 5′-TAT GAG CCA GAA GAA CTT TTA GGC-3′ and antisense 5′-CAC CTC TTT TGG CAA GCA TCC TG-3′ for HIF-1α; sense 5′-CTG TGT CTG AGA AGA GTA ACT TCC-3′ and antisense 5′-TTG CCA TAG GCT GAG GAC TCC T-3′ for HIF-2α; sense 5′-ACT GCT GGC AGA CAT GGG GA-3′ and antisense 5′-GCT GCA AGT TGG AGA TCT GCT TC-3′ for keratin 1; sense 5′-CAA CTG GCC TTG AAA CAA TC-3′ and antisense 5′-TCT GTT GCA ACT GTT CTT CC-3′ for keratin 10; sense 5′-ACA ACT TTG GTA TCG TGG AAG G-3′ and antisense 5′-GCC ATC ACG CCA CAG TTT C-3′ for GAPDH. Amplification was performed for 45 cycles. Each cycle consisted of denaturation at 95°C for 30 s, annealing at 55°C for 30 s, and extension at 72°C for 20 s. The mRNA levels were determined by calculating 2^-∆∆Ct^ values.

### Western blot analysis

The protein amounts of Iκ-Bα, NF-κB, p38, JNK, and EGF receptor and their phosphorylated forms were quantified with specific antibodies in western blotting. The cultures were lysed, and the lysates were dissolved in lysis buffer containing 0.125 M Tris-HCl, pH 6.8, 4% SDS, 20% glycerol (Thermo Fisher Scientific), 2% 2-mercaptoethanol and 0.02% bromophenol blue, and denatured at 95°C for 5 min, three times. The reduced samples were normalized to 40 µg per lane and separated in 10% sodium dodecyl sulphate (SDS)-polyacrylamide gels, run at 60–100 V and then transferred to nitrocellulose membranes. The membrane was blocked with 5% reduced fat milk, or 5% BSA, for 1 h at room temperature. After blocking, the membrane was incubated with primary antibodies overnight at 4°C. Primary antibodies used: mouse anti-phospho-Iκ-Bα at 1:500 (Novus Biologicals, Centennial, CO), mouse anti-Iκ-Bα at 1:500 (Novus Biologicals), rabbit anti-phospho-NF-κB at 1:1,000 (Cell Signaling Technology), rabbit anti-NF-κB at 1:1,000 (Santa Cruz Biotechnology, Dallas, TX), rabbit anti-phospho-p38 MAPK (Thr180/Tyr182) antibody at 1:1,000 (Cell Signaling Technology, Danvers, MA), rabbit anti-p38 MAPK antibody at 1:1,000 (Cell Signaling Technology), mouse anti-phospho-SAPK/JNK (Thr183/Tyr185) antibody at 1:1,000 (Cell Signaling Technology), rabbit anti-SAPK/JNK antibody at 1:1,000 (Cell Signaling Technology), rabbit anti-phospho-EGF receptor (Tyr1068) at 1:1,000 (Cell Signaling Technology), rabbit anti-EGF receptor at 1:1,000 (Santa Cruz Biotechnology). The membranes were followed by secondary antibodies having incubation for 2 h at room temperature, where HRP-conjugated antibodies at 1:2,000 (Zymed, South San Francisco, CA) were used. Non-specific binding of protein was reduced with 0.1% Tween-20 TBST (pH 7.6) (Anatrance, Maumee, OH). The enhanced chemiluminescence (ECL) western blotting detection kit (Thermo Fisher Scientific; peroxide solution, luminol enhancer solution) was used. The amount of protein was compared with the band intensities, measured under ChemiDoc Imaging System (Bio-Rad Laboratories).

### Immunofluorescent staining

HaCaT keratinocytes were seeded on a sterile coverslip (Marienfeld Superior, Lauda-Königshofen, Germany) in 12-well plate at 5 × 10^4^ cells/mL. After the treatment, cultured keratinocytes were fixed with 4% paraformaldehyde for 10 min at room temperature, followed by blocking with 5% BSA in PBS with permeabilization by 0.1% Triton for 2 h at room temperature. After blocking, the culture was incubated with primary antibodies, anti-phospho-NF-κB p65 at 1:100 (Santa Cruz Biotechnology) overnight at 4°C. The samples were followed by secondary antibodies having incubation for 2 h at room temperature under darkness: 647 (donkey anti-rabbit IgG)-conjugated antibody at 1:200 (Abcam Ltd.) was used. The samples were mounted with ProLong Gold Antifade Mountant with DAPI (Thermo Fisher Scientific). Samples were then examined by a Leica SP8 Confocal Microscope (Leica Microsystems, Wetzlar, Germany) with a 63x oil immersion objective.

### Animals

C57BL/6 mice were supplied by Animal and Plant Care Facility in The Hong Kong University of Science and Technology (HKUST). All experiments were performed according to the guidelines of Department of Health, The Government of Hong Kong SAR. The experimental procedures had been reviewed and approved by the Animal Ethics Committee of the university (Reference No, (20–104) in DH/HT&A/8/2/2 Pt.2). Housing was maintained at a constant temperature and humidity, under a fixed 12-h light/dark cycle and free access to food and water.

### Induction of dermatitis-like skin lesion in mice

C57BL/6 mice (2- to 3-month-old) were randomly divided into six groups as follows: control group (vehicle-treated), DNCB (2,4-dinitrochlorobenzene) group (challenged with 7% w/w DNCB cream on day 1 and day 6), dexamethasone group (challenged as DNCB group and treated with 0.12% w/w dexamethasone cream), NANA group (challenged as DNCB group and treated with 1, 10, 100 mM NANA cream), and EBN extract/digest group (challenged as DNCB group and treated with 0.1, 1, 10 mg/mL EBN extract/digest cream). Vaseline was used as a vehicle. On day 0, the experimental mice were anesthetized by inhalation of isoflurane, and the dorsal skin of each mouse was shaved for topical application of cream. Mice from all groups, except control group, were challenged with 7% w/w DNCB cream on day 1 and day 6, followed by subsequent treatment of dexamethasone/NANA/EBN extract/EBN digest creams. Mice were sacrificed by cervical dislocation on day 11. The animal model was designed in slight modification based on previous reports (Wang et al., 2018; Wang et al., 2021; Guo et al., 2022).

### Frozen *ex vivo* mouse skin section

The dorsal skin was collected from C57BL/6 mice after sacrificed by cervical dislocation. The isolated skin was rinsed with cold PBS and fixed with 4% paraformaldehyde for 2 h at room temperature, followed by dehydrating with 30% sucrose in PBS overnight at 4°C. After dehydration, the skin was embedded in optimal cutting temperature (OCT) compound in a tissue mold, followed by an overnight freezing under -80°C. Skin was then cut into 10 and 50 µm thick sections at -20°C using CryoStar™ NX70 Cryostat (Thermo Fisher Scientific).

### Hematoxylin and eosin staining

The mouse dorsal skin sections were stained with hematoxylin and eosin (H&E) staining kit, purchased from Abcam. The sections were first washed with PBS twice for 5 min and dried, before hematoxylin staining for 5 min. The hematoxylin-stained sections were washed with DDI water, twice. The incubation with bluing reagent was performed for 10–15 min, and the sections were then washed with DDI water, twice, followed by absolute ethanol. Incubation with eosin solution was subsequently performed for 3 s, and dehydration with absolute ethanol was conducted. Sample was then mounted with dibutyl phthalate in xylene reagent (Sigma-Aldrich) for microscopic evaluation. The samples were examined by a Zeiss Axio Vert. A1 Inverted phase microscope (Zeiss, Jena, Germany) with a 10x objective.

### Toluidine blue staining

The mouse dorsal skin sections were stained with the toluidine blue O purchased from Macklin (Shanghai, China). Sections were first washed with PBS twice for 5 min and dried, before incubation in 0.1% toluidine blue staining solution in 1% NaCl solution, pH 2.3, for 5–30 min. Toluidine blue-stained sections were washed with DDI water, twice for 5 min. Differentiation of nuclei and particles were followed by 0.5% glacial acetic acid. Rapid dehydration was subjected with 95% absolute ethanol, followed by dibutyl phthalate in xylene reagent (Sigma-Aldrich) for microscopic evaluation.

### ELISA

Mouse dorsal skin were collected and weighed for 30–100 mg. Total proteins were extracted by 200 μL RIPA buffer. Terminal blood collection was performed via cardiac puncture. Serum was obtained by centrifugation at 2,000 rcf, 20 min at 4°C, after sitting at room temperature for 2 h. Amounts of IL-1β, IL-6, TNF-α, FLG and serum IgE were quantified by using enzyme-linked immunosorbent assay (ELISA). The microtiter plates were pre-coated with monoclonal mouse antibodies of anti-IL1β (Novus Biologicals), anti-IL-6 (Novus Biologicals), anti-TNF-α (R&D Systems, Minneapolis, MN), anti-FLG (G-Biosciences, St. Louis, MO), and anti-IgE (G-Biosciences), respectively. Absorbance was determined at 450 nm, with a background correction at 570 nm.

### Statistical analysis

Data for control and drug-treated groups were compared using one-way ANOVA with Dunnett post-hoc statistical testing provided in GraphPad Prism 8.3.0. Results were calculated from at least three independent determinations, performed in triplicates, and expressed as percent change, percent increase or fold change of control in mean ± SEM. Statistical significance was indicated by *p < 0.05, **p < 0.01 and ***p < 0.001.

## Results

### EBN suppresses TNF-α-induced inflammatory responses

**FIGURE 1:**
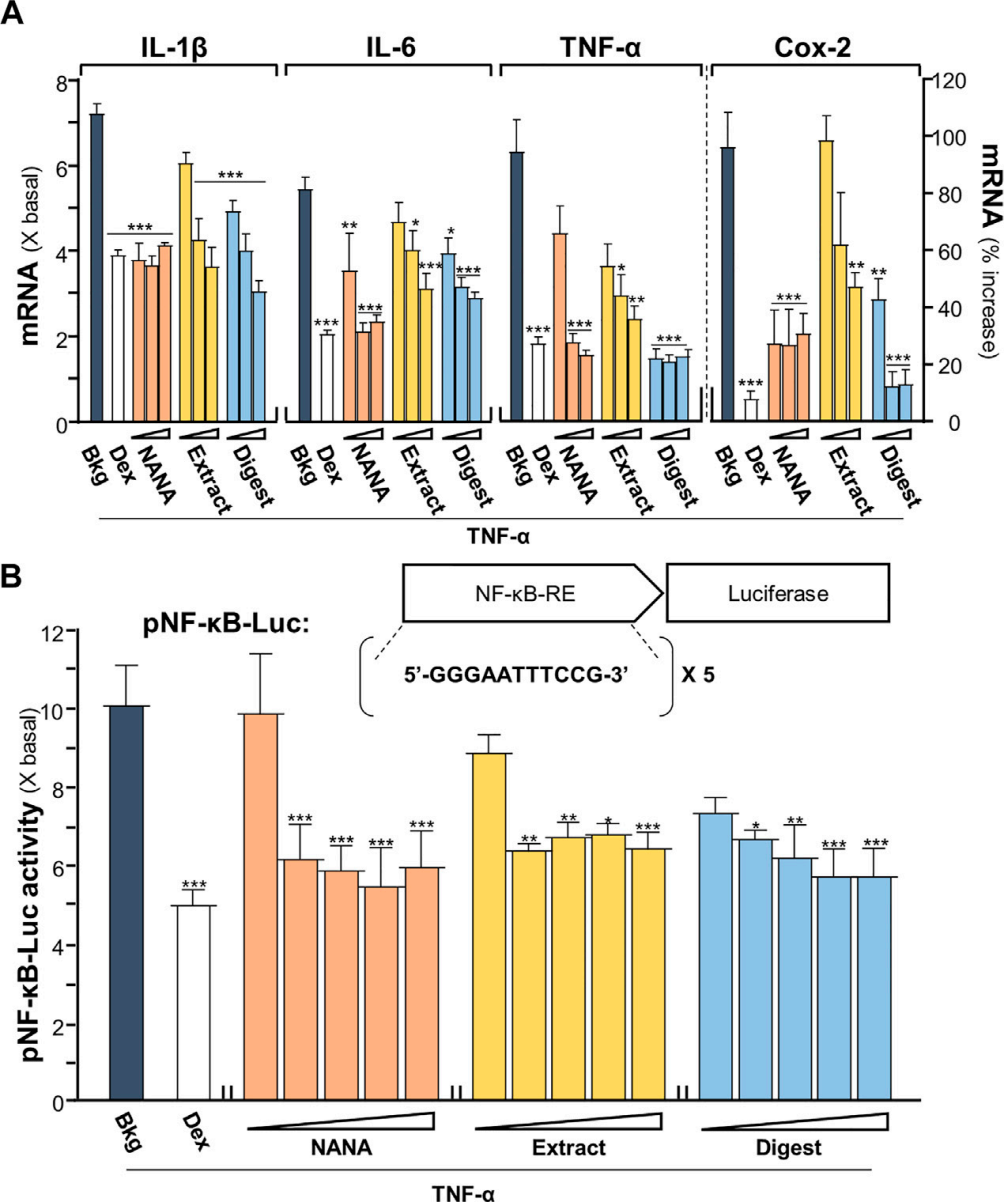
EBN reduces the TNF-α-induced inflammatory responses. **(A)** The mRNA levels of IL-1β, IL-6, TNF-α and Cox-2 in cultured HaCaT keratinocytes after 2-h co-treatment of TNF-α (20 ng/mL) and EBN preparations are shown. NANA (0.05, 0.1, 1 mM), and EBN extract or digest (1, 10, 100 μg/mL) were applied, as indicated. Dexamethasone at 10 nM was a positive control. **(B)** The pNF-κB-Luc plasmid, composed of 5 NF-κB response elements tagged with firefly luciferase reporter, was transfected into keratinocytes for 24 h, followed by TNF-α (20 ng/mL) stimulation and EBN incubation for 2 h. The doses were NANA (0.01, 0.05, 0.1, 0.5, 1 mM), and EBN extract or digest (1, 5, 10, 50, 100 μg/mL). The luciferase activity of each well was normalized to total protein. The values are expressed, except Cox-2 mRNA level shown in percentage of increase, as the fold change to normalized basal activity set at 1, in mean ± SEM, *n* = 6. Statistically significant results are marked with *p < 0.05, **p < 0.01 and ***p < 0.001 against the TNF-α-treated group.

An inflammatory model of cultured keratinocytes was established by using applied TNF-α. In TNF-α-treated keratinocytes, the amounts of mRNAs encoding inflammatory cytokines, e.g., IL-1β, IL-6 and TNF-α, were markedly increased by 5 to 7 folds (Figure 1A). In parallel, the mRNA encoding Cox-2, an inducible form of cyclo-oxygenase catalyzing the conversion of arachidonic acid to prostaglandins, was induced by applied TNF-α by over 100% of increase. The results suggested the proper establishment of inflammatory cell model. Two forms of EBN, e.g., EBN extract and EBN digest, as well as its major ingredient NANA, were tested here for their anti-inflammatory responses in the cultures. The chemical characterizations of EBN extract and digest have been described (Lai et al., 2021). The amount of NANA within EBN extract/digest was determined by LC-MS, while HPLC profiles using gel filtration and reverse phase columns were shown for EBN extract/digest (Supplementary Figures S1A-D). In addition, EBN extract or digest did not show cytotoxicity up to 500 μg/mL (Supplementary Figure S1E). Under all scenarios, the mRNA expressions of IL-1β, IL-6, TNF-α and Cox-2 were markedly reduced in TNF-α-treated keratinocytes after treating with EBN, and the suppression was in a dose-dependent manner (Figure 1A). The maximal suppression, as revealed in Cox-2 expression, could be at ∼80%. By comparison, NANA or EBN digest was consistently better than that of EBN extract (Figure 1A).

A DNA construct having 5X NF-κB response elements tagged with a downstream luciferase reporter, named as pNF-κB-Luc, was employed here in the plasmid transfecting cell model. In pNF-κB-Luc transfected keratinocytes, the luciferase signal, representing the promoter activity, was robustly induced to ∼10 folds by TNF-α (Figure 1B). The amount of luciferase activity was significantly diminished, at least by 50%, after the co-treatment of NANA, EBN extract or EBN digest, in a dose-dependent manner. This result was in line to the outcomes of cytokine and Cox-2 analyses under the EBN treatment.

**FIGURE 2:**
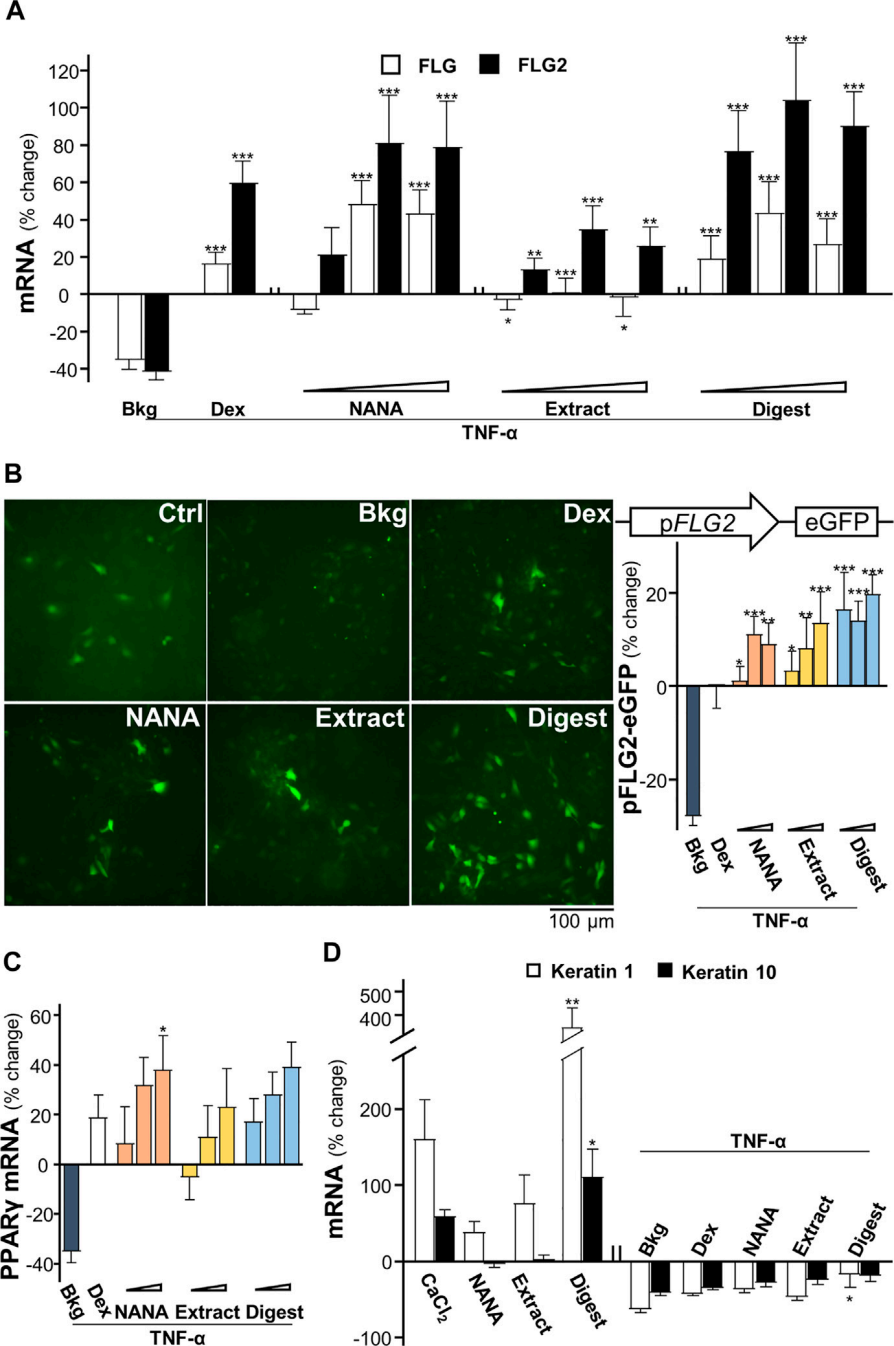
EBN restores the TNF-α-reduced expressions of filaggrin and filaggrin-2 via induction of moisturization and differentiation. **(A)** The mRNA levels of filaggrin and filaggrin-2 in cultured HaCaT keratinocytes after 2-h co-treatment of TNF-α (20 ng/mL) and EBN preparation are shown. NANA (0.05, 0.1, 1 mM), and EBN extract or digest (1, 10, 100 μg/mL) were applied, as indicated. Dexamethasone at 10 nM was a positive control. **(B)** The pFLG2-eGFP plasmid, composed of the vector pEGFP-N1 containing a filaggrin-2 promoter, was transfected into keratinocytes for 24-h, followed by seeding at 1 × 10^5^ cells/mL in a 96-well black clear bottom plate. A 24-h pre-incubation of EBN, followed by another 24-h stimulation of TNF-α (100 ng/mL). The doses were as in (A). Representative fluorescence images are displayed, with doses of 1 mM and 100 μg/mL selected for NANA and EBN extract or digest, respectively (left panel). The GFP activity of each well was normalized to total protein (right panel). **(C)** The mRNA levels of PPARγ in cultured HaCaT keratinocytes after 2-h co-treatment of TNF-α (20 ng/mL) and EBN preparation (right panel), are shown. The doses were as in (A). **(D)** The mRNA levels of keratin 1 and keratin 10 in cultured HaCaT keratinocytes are shown. NANA (1 mM), EBN extract and digest (both at 100 μg/mL) were applied for 24-h, or simultaneously with TNF-α (20 ng/mL) for 2-h. CaCl_2_ (0.16 mM) and dexamethasone (10 nM) were positive controls. Values are expressed as the percentage of change relative to normalized basal expression set at 0%, in mean ± SEM, *n* = 6. Statistically significant results are marked with *p < 0.05, **p < 0.01 and ***p < 0.001 against the control, or the TNF-α-treated group, depending on experiments performed without or with TNF-α.

The mRNAs encoding filaggrin and filaggrin-2 have been shown to be induced by EBN extract and digest in cultured keratinocytes (Lai et al., 2021). In addition, application of NANA induced such mRNA expressions (Supplementary Figure S2), which showed an induction up to 150% increase in expression of filaggrin-2. Disruption of genes expressing epithelial structural proteins, e.g., filaggrin, has been identified in AD patients (Ghosh, et al., 2015). To determine whether EBN rescuing the TNF-α-mediated gene expressions, the transcriptional levels of filaggrin and filaggrin-2 were analyzed by RT-PCR. Under TNF-α challenge, the mRNA levels of filaggrin and filaggrin-2 were reduced by ∼40% (Figure 2A). Having the application of NANA and EBN digest in the cultures, the expressions of filaggrin and filaggrin-2 were markedly induced. The induction was achieved in a dose-dependent manner. The expression of filaggrin-2 was highly sensitive to the treatment, having the maximal induction at ∼100% increase under the effects of NANA and EBN digest. The induction by EBN extract was not that robust, as compared to NANA or EBN digest (Figure 2A). In parallel, the result was further validated by the GFP-tagged promoter of filaggrin-2, i.e., pFLG2-eGFP, in DNA transfected keratinocytes. Under TNF-α challenge, the promoter activity was reduced by ∼30%. The treatments with NANA, EBN extract and EBN digest recovered the diminished promoter activity (Figure 2B). Dexamethasone served as a control.

The nuclear receptor, peroxisome proliferator-activated receptor PPARγ, is inhibited by TNF-α predominantly via activation of IKK/NF-κB signaling pathway (Ye, 2008). The EBN-mediated regulation of filaggrin and filaggrin-2 has been shown to be triggered by PPARγ (Lai et al., 2021). In parallel, NANA showed the mRNA induction in a dose-dependent manner (Supplementary Figure S2). In the presence of TNF-α, the mRNA level of PPARγ was inhibited by ∼40% (Figure 2C). Applying NANA and EBN preparation reversed the inhibited mRNA levels of PPARγ in dose-dependent manners (Figure 2C).

According to Rabeony et al. (2014), the challenge of TNF-α keratinocytes resulted in ∼75-fold decrease of keratin 1 mRNA, but not on the expression of keratin 10. In view of the inhibition of keratinocyte differentiation exhibited by TNF-α, the effects of NANA and EBN preparation on the differentiation markers, keratin 1 and keratin 10 (Sanz-Gomez et al., 2020), were determined. EBN digest induced a maximal increase to ∼400% for keratin 1 and ∼100% for keratin 10 (Figure 2D). Under TNF-α stimulation, the mRNA levels of keratin 1 and keratin 10 were inhibited by ∼50%. Applying NANA and EBN preparation reversed the inhibited mRNA levels of keratin 1 and keratin 10 (Figure 2D).

### EBN regulates inflammatory signaling

**FIGURE 3:**
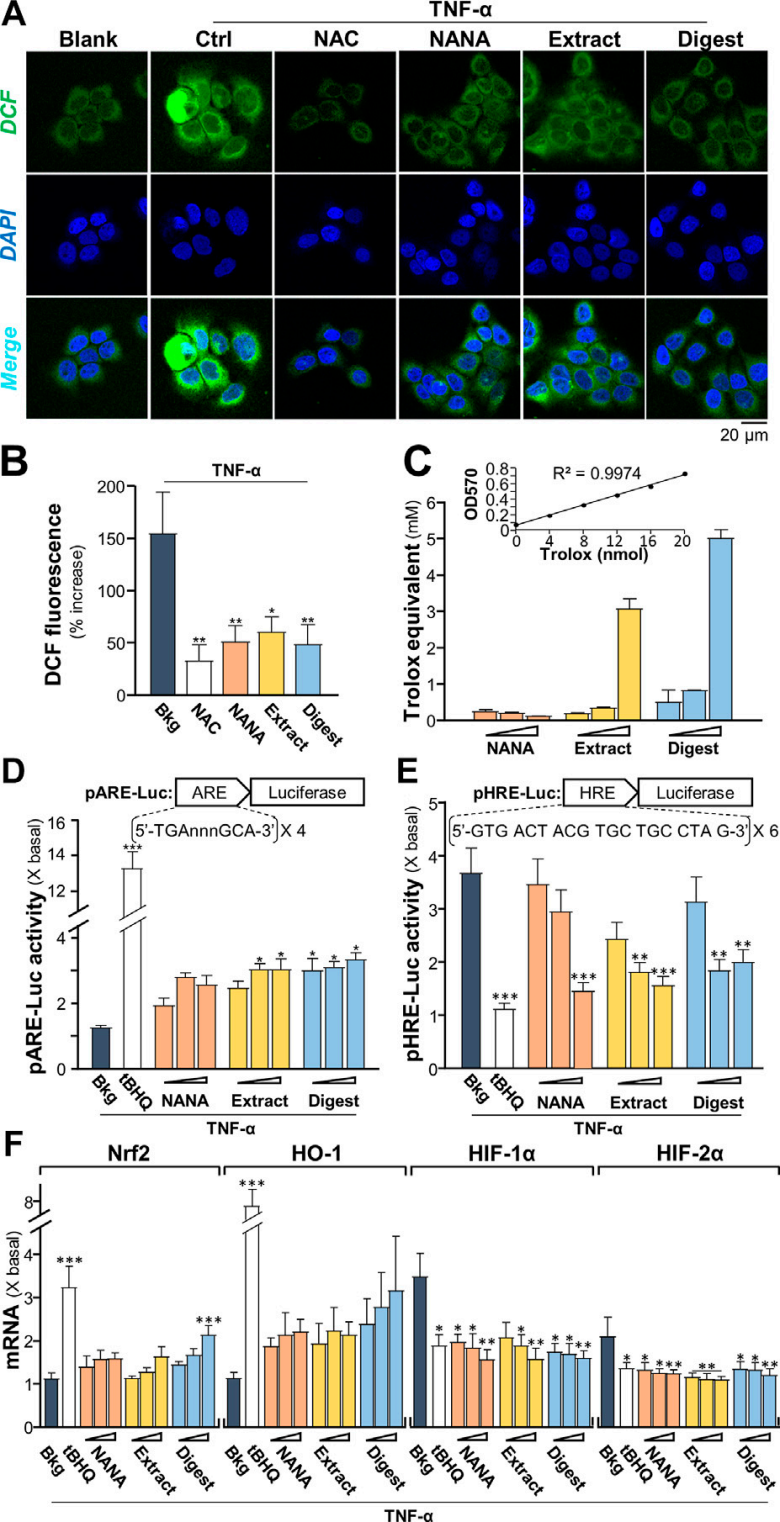
EBN suppresses the formation of TNF-α-stimulated reactive oxygen species. **(A)** Cytosolic ROS formation in HaCaT keratinocytes is indicated by DCF in representative confocal images. Cultured keratinocytes were incubated with or without EBN for 2-h, followed by 5 min TNF-α (20 ng/mL) stimulation, and stained by DCFH-DA for 20 min. One-hour pre-incubation of 20 mM NAC, a known ROS scavenger, was a positive control. Representative fluorescence images are displayed. NANA at 1 mM, EBN extract/digest (100 μg/mL) are shown. **(B)** The quantifications from (A) are expressed as the percentage increase in fluorescence intensity to normalized basal activity set at 0%. **(C)** Total antioxidant capacity of NANA (0.05, 0.1, 1 mM) and EBN extract or digest (1, 10, 100 μg/mL), as indicated, were measured for OD_570_. **(D)** pARE-Luc or **(E)** pHRE-Luc plasmids, each tagged with a firefly luciferase reporter, was transfected into keratinocytes for 24-h, followed by TNF-α (20 ng/mL) stimulation and EBN incubation for 6 h, respectively. **(F)** The mRNA levels of Nrf2, HO-1, HIF-1α and HIF-2α in cultured HaCaT keratinocytes after 6-h co-treatment of TNF-α (20 ng/mL) and EBN preparation are shown. The doses were as in **(C)**. Tert-butylhydroquinone (tBHQ) at 20 μM was a positive control. Values are expressed as the fold change to normalized basal activity set at 1. All values are in mean ± SEM, *n* = 6. Statistically significant results are marked with *p < 0.05, **p < 0.01 and ***p < 0.001 against the TNF-α-treated group.

Intracellular reactive oxygen species (ROS) is induced by TNF-α, which augments the signaling as a feedback control (Blaser, et al., 2016). To identify the TNFα-induced ROS generation, 2′-7′ dichlorofluorescin diacetate (DCFH-DA), a fluorogenic dye could be oxidized by ROS into green fluorescent, 2′-7′ dichlorofluorescin (DCF), was used to measure ROS level in cultured keratinocytes under treatments of EBN. The TNF-α-induced DCF green fluorescent was markedly reduced, at least by ∼50%, by the presence of NANA, EBN extract and EBN digest (Figures 3A,B). N-acetyl cysteine (NAC), a known ROS scavenger, was a positive control here. The ROS scavenging ability of EBN could be accounted partly for its antioxidant property. In total antioxidant assay, EBN extract and digest showed robust Trolox equivalent of antioxidative activities (Figure 3C). Here, NANA showed no effect.

As depicted by Blaser et al. (2016), the TNF-α-induced NF-κB signaling could lead to up regulation of antioxidants via Nrf2-ARE pathway, as repressed by NF-κB p65-KEAP1 interaction and NF-κB co-activator MafK. Here, a promoter of antioxidant response element (ARE), tagged downstream with a luciferase reporter, i.e., pARE-Luc, was adopted to assess the role of EBN in regulating antioxidation. In pARE-Luc transfected keratinocytes, the luciferase signal, representing the promoter activity, was slightly induced by TNF-α (Figure 3D). The promoter activity was increased markedly by NANA, EBN extract and EBN digest by two to three folds. A known Nrf2 activator, tert-butylhydroquinone (tBHQ), was adopted as a positive control to activate the promoter.

As demonstrated by Haddad and Land (2001), the normoxic translocation/activation of HIF-1α, stimulated by TNF-α, is mediated by a ROS-dependent mechanism. The transcriptionally active HIFs required a heterodimerization between HIF-α and HIF-1β, which activated the hypoxia response element (HRE) to transcribe the hypoxia modulated genes. Here, a promoter of HRE, tagged downstream with a luciferase reporter, i.e., pHRE-Luc, was adopted to assess the role of EBN in regulating HIF binding activity under TNF-α challenge. In pHRE-Luc transfected keratinocytes, the luciferase signal, representing the promoter activity, was induced ∼3.5 folds by TNF-α (Figure 3E). The promoter activity was markedly reduced to ∼1.5 – 2-fold by NANA, EBN extract and EBN digest. tBHQ was a positive control to repress the promoter (Figure 3E). Since the ROS signaling induced by TNF-α could activate Nrf2-and HO-1-mediated pathways (Blaser et al., 2016). TNF-α induces the levels of HIF-1β via activation of IKK-dependent NF-κB signaling pathway, which indirectly regulates the levels of HIF-α (Van Uden et al., 2011). To probe the role of EBN preparation in counteracting the TNF-α induced ROS- or IKK/NF-κB- mediated gene expression, the transcriptional levels of Nrf2, HO-1, HIF-1α and HIF-2α in the treated cultures were analyzed by RT-PCR. Under TNF-α challenge, the mRNA levels of Nrf2 and HO-1 were slightly induced (Figure 3F). Having the application of EBN digest in the cultures, the expressions of Nrf2 and HO-1 were promoted by ∼2 folds. On the other hand, the TNF-α-stimulated expressions of HIF-1α and HIF-2α were significantly suppressed by the treatments. The treatments with NANA, EBN extract and EBN digest reduced the activated expressions of HIF transcription factors in dose-dependent manners (Figure 3F).

**FIGURE 4:**
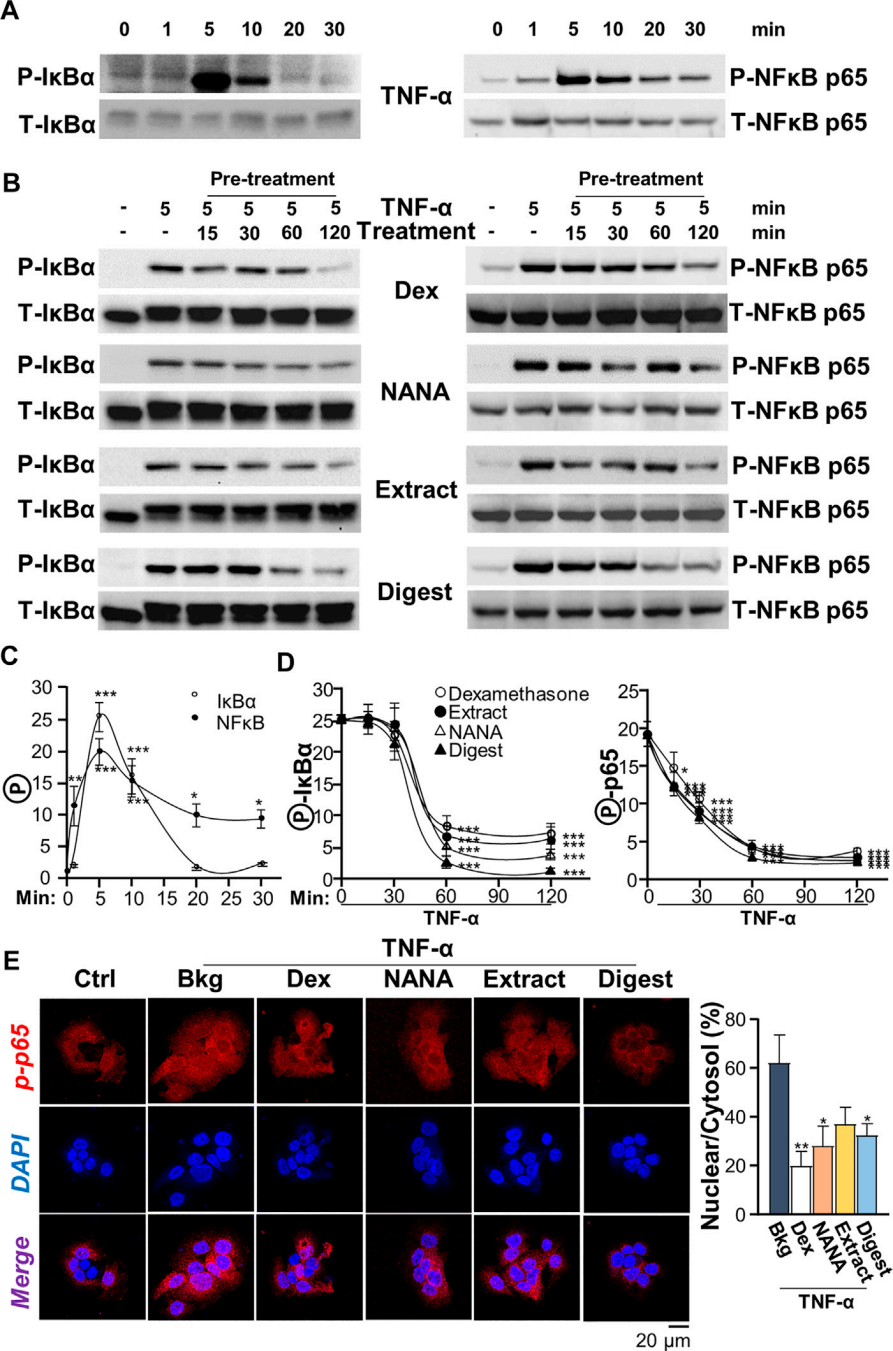
EBN inhibits TNF-α-induced phosphorylations of Iκ-Bα and NF-κB p65. **(A)** After 16-h serum starvation, cultured keratinocytes were induced by TNF-α (20 ng/mL) at a period of 0–30 min, as indicated. Total Iκ-Bα and NF-κB p65 (T-Iκ-Bα, 38 kDa and T-NF-κB p65, 65 kDa) and phosphorylated Iκ-Bα and NF-κB p65 (P-Iκ-Bα, 38 kDa and P-NF-κB p65, 65 kDa) were evaluated by western blot assays. **(B)** After 16-h serum starvation, EBN pre-treatment in cultured keratinocytes were tested. Pre-incubation at 15, 30, 60 and 120 min was performed. Dexamethasone (a positive control, 10 nM), NANA (1 mM), EBN extract and digest (both at 100 μg/mL) were applied, and then cells were incubated with TNF-α for 5 min afterwards. **(C)** The quantifications of P-Iκ-Bα/T-Iκ-Bα and P-NF-κB p65/T-NF-κB p65 expression levels after different time of TNF-α treatment are shown. **(D)** The quantifications of P-Iκ-Bα/T-Iκ-Bα and P-NF-κB p65/T-NF-κB p65 expression levels from pre-treatments of **(B)** are shown. **(E)** Cytosolic and nuclear expressions of phosphorylated NF-κB p65 protein in keratinocytes after 4-h pre-treatment of NANA (1 mM), EBN extract and EBN digest (both at 100 μg/mL) and 5 min TNF-α stimulation are shown, in representative confocal images (left panel). The quantifications of P-NF-κB p65 nuclear to cytosol intensity percentage under different treatments are shown (right panel). Values are expressed as the fold change to normalized basal activity set at 1, in mean ± SEM, *n* = 3. Statistically significant results are marked with *p < 0.05, **p < 0.01 and ***p < 0.001 against the TNF-α-treated group.

In cultured keratinocytes, the phosphorylations of Iκ-Bα and NF-κB p65, activated by TNF-α, were measured by using western blot analysis. The maximal phosphorylations of Iκ-Bα and NF-κB p65 in responding to TNF-α challenge were transient and peaked at ∼5 min (Figures 4A,B). The ratios of P-Iκ-Bα/T-Iκ-Bα and P-NF-κB p65/T-NF-κB p65 reached ∼25- and ∼20-folds, respectively (Figure 4C). To study the ability of EBN in phosphorylation, the responding time from 15 to 120 min of pre-treatment with EBN was monitored. In the cultures, NANA, EBN extract and digest showed robust suppression of the phosphorylations, i.e. P-Iκ-Bα and P-NF-κB p65, in time-dependent manners, and the maximum suppression was at 1 h of treatment (Figures 4B,D). Both NANA and EBN digest showed the best suppressing response. In addition, the nuclear translocation of p-NF-κB p65 was assessed by immunostaining in cultured keratinocyte. By quantification of nuclear to cytosol ratio of p-NF-κB p65 staining intensity, NANA showed the most significant inhibition at ∼50% reduction (Figure 4E). Dexamethasone served as a positive control in all assays.

**FIGURE 5:**
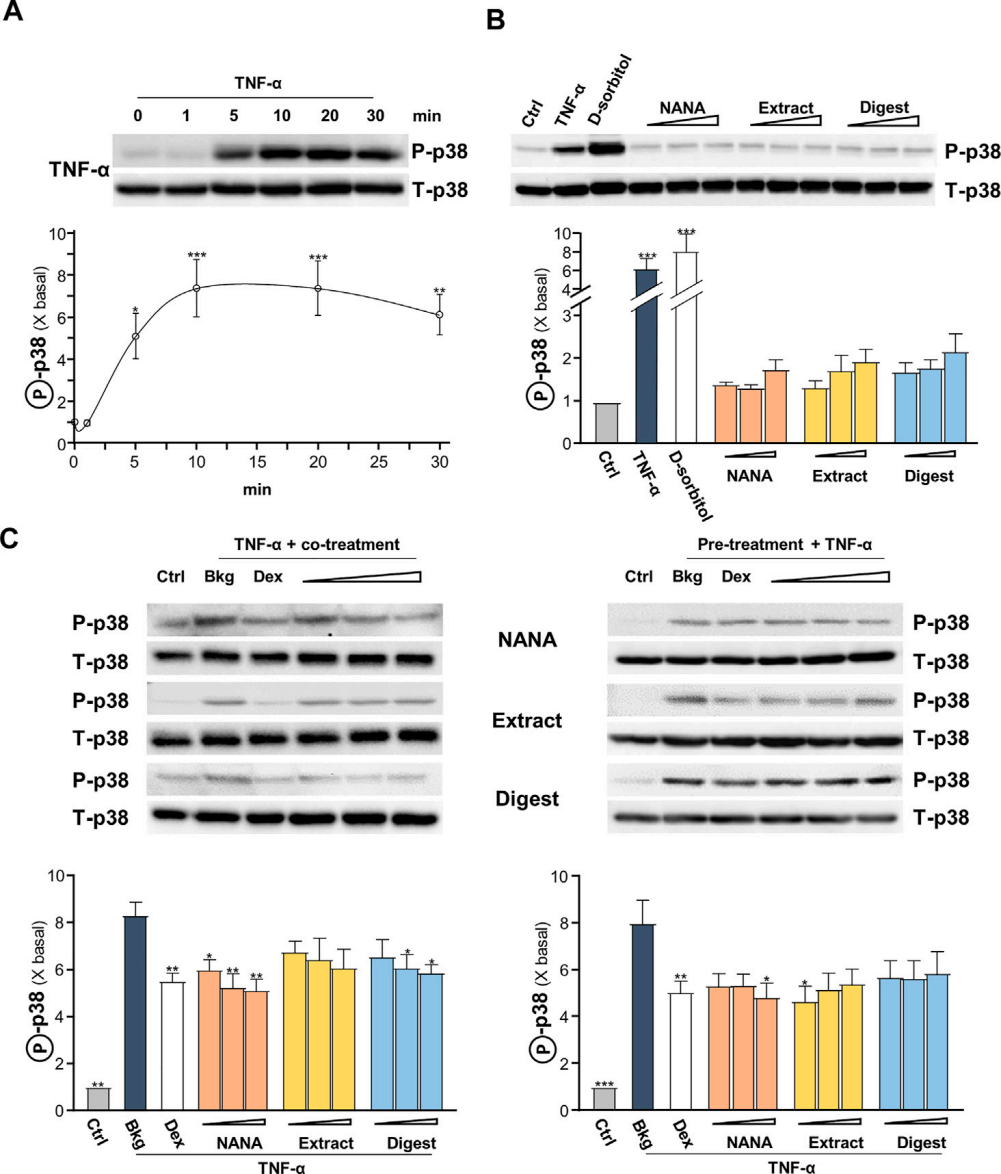
EBN inhibits TNF-α-induced p38-MAPK phosphorylation. After 16-h serum starvation, cultured keratinocytes were: **(A)** induced by TNF-α (20 ng/mL) at a period of 0–30 min, as indicated; or **(B)** treated with EBN preparation alone for 1-h, TNF-α (20 ng/mL), D-sorbitol (a positive control, 400 mM), NANA (0.05, 0.1, 1 mM), EBN extract and digest (both at 1, 10, 100 μg/mL) are indicated. **(C)** After 16-h serum starvation, (left panel) simultaneous incubation of TNF-α together with EBN preparation for 5 min was performed; (right panel) 1-h pre-treatment of EBN preparation followed by 5 min TNF-α stimulation. The doses were as in **(B)**. Dexamethasone at 10 nM was a positive control. Total p38-MAPK (p38-MAPK) and phosphorylated p38-MAPK (p-p38 MAPK) (both at ∼38 kDa) were evaluated by western blot assays. The values of P-p38/T-p38 under different conditions are expressed as the fold change to normalized basal activity set at 1, in mean ± SEM, *n* = 3. Statistically significant results are marked with *p < 0.05, **p < 0.01 and ***p < 0.001 against the control, or the TNF-α-treated group in **(C)**.

The signaling of MAPK is known to positively regulate NF-κB signaling (Wang et al., 2009). Thus, the TNF-α-induced p38 MAPK phosphorylation was determined here. In cultured keratinocytes, applied TNF-α induced p38 phosphorylation in a time-dependent manner, having a maximal induction at ∼8 folds after 10 min of challenge: the phosphorylation was maintained after 30 min (Figure 5A). By our previous study, EBN preparation could induce p38-MAPK phosphorylation (Lai et al., 2021). In the cultures, applications of NANA and EBN preparation for 1 h were able to induce slightly the p38 phosphorylation (Figure 5B). TNF-α boosted ∼6 folds of increase after the application, approximating to the effect of a positive control D-sorbitol. The p38 phosphorylation, triggered by TNF-α, was suppressed in simultaneously applied NANA and EBN extract/digest at 5 min (Figure 5C, left). The level of total p38 was not changed in all situations. In addition, the pre-treatments of NANA and EBN extract/digest in cultures could also suppress the TNF-α-induced p38 phosphorylation (Figure 5C, right).

**FIGURE 6:**
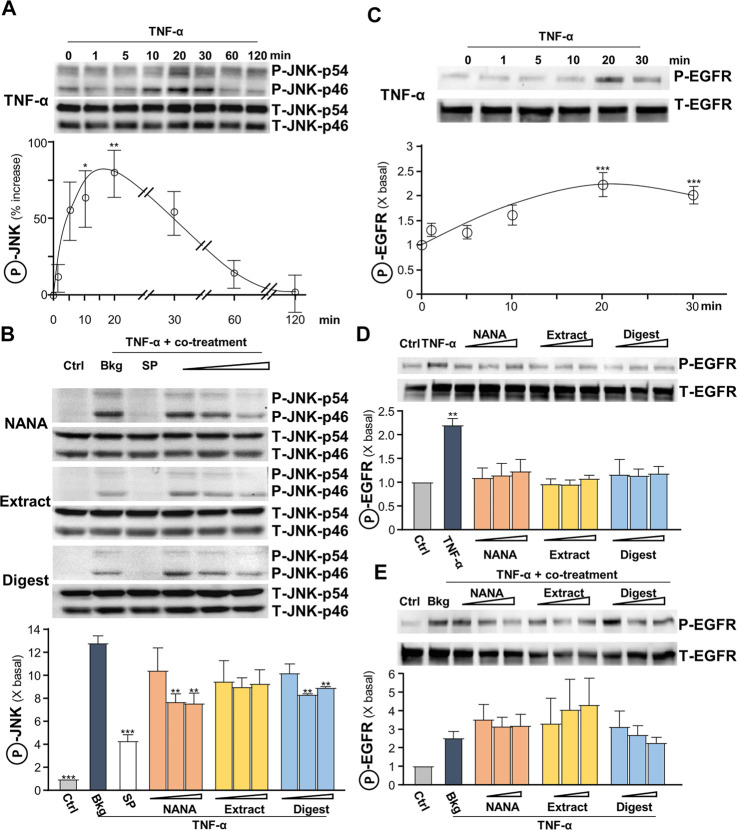
EBN inhibits TNF-α-induced JNK phosphorylation, while shows no effect in TNF-α-induced EGF receptor phosphorylation. **(A)** After 16-h serum starvation, cultured HaCaT keratinocytes were induced by TNF-α (20 ng/mL) at a period of 0–2 h. The values of p-JNK/JNK expression levels at different time points are shown. **(B)** After 16-h serum starvation, TNF-α together with EBN preparation were applied for 20 min. SP600125 (a JNK inhibitor; 10 nM), NANA (0.05, 0.1, 1 mM), EBN extract and digest (both at 1, 10, 100 μg/mL) are indicated. Total JNK (T-JNK-p46 and T-JNK-p54 for isoforms at 46 and 54 kDa respectively) and phosphorylated JNK (P-JNK-p46 and P-JNK-p54 for isoforms at 46 and 54 kDa, respectively) were evaluated by western blot assays. **(C)** After 16-h serum starvation, cultured HaCaT keratinocytes were induced by TNF-α (20 ng/mL) at a period of 0–30 min. The values of p-EGFR/EGFR expression levels at different time points are shown. After 16-h serum starvation, cultured HaCaT keratinocytes were incubated with EBN preparation for 20 min **(D)** without or **(E)** with TNF-α (20 ng/mL) simultaneously. Total EGFR (at 170 kDa) and phosphorylated EGFR (at 175 kDa) were evaluated by western blot assays. The values of P-JNK/T-JNK and P-EGFR/T-EGFR under different conditions are expressed as the fold change to normalized basal activity set at 1, in mean ± SEM, *n* = 3. Statistically significant results are marked with *p < 0.05, **p < 0.01 and ***p < 0.001 against the control in **(D)**, or the TNF-α-treated group.

The signaling of c-Jun N-terminal kinase (JNK), or stress-activated protein kinase (SAPK), was reported to be involved in regulating the levels of ROS and filaggrin (Blaser et al., 2016). In addition, TNF-α was reported to inhibit the expression of filaggrin through JNK (Kim et al., 2011). Thus, the role of EBN in JNK signaling, i.e., regulating ROS and filaggrin expression, in TNF-α-insulted keratinocytes was determined. The maximal TNF-α-induced JNK phosphorylation, both at p46 and p54 having ∼70% increase, was at transient manner having maximum at 10–20 min (Figure 6A). NANA, EBN extract/digest suppressed the phosphorylation of JNK, and again NANA and EBN digest showed the best responses (Figure 6B). The specific JNK inhibitor SP600125 served as a positive control to block the induced phosphorylation.

Leukocyte-derived cytokines, including TNF-α, were reported to be potent inducers of epidermal growth factors (EGF) and receptor (EGFR), which attributed to epidermal hyperplasia and thickening in chronic skin disorders (Pastore et al., 2008). Yet, EGFR blockade may aggravate skin inflammatory response, while active EGFR signaling may favor anti-inflammatory condition. A transient response of TNF-α-mediated EGFR phosphorylation was peaked at 20 min (Figure 6C). EBN was reported to contain EGF-like activity (Kong et al., 1987), and therefore the ability of EBN to phosphorylate EGFR was tested. NANA and EBN preparation did not activate EGFR phosphorylation (Figure 6D), as well as not able to suppress the phosphorylation triggered by TNF-α (Figure 6E).

### EBN relieves dermatitis in mouse skin

**FIGURE 7:**
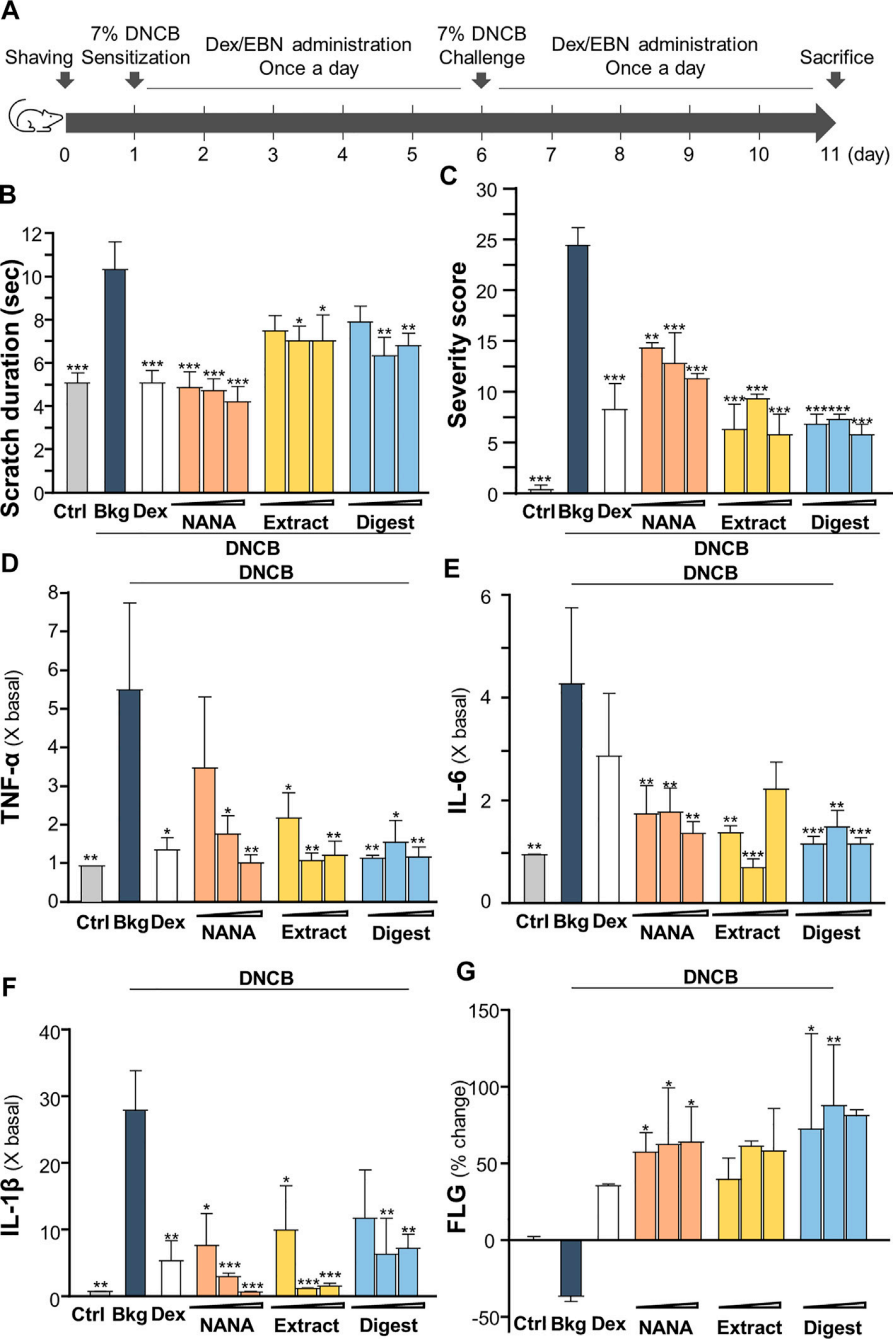
EBN ameliorates the symptoms of DNCB-induced atopic dermatitis mice. **(A)** Time course of EBN treatment in the DNCB-induced mice model is shown. The mice were acclimatized and shaved 1 day before chemical sensitization by 7% DNCB on dorsal skin. NANA (1, 10, 100 mM), EBN extract and digest (0.1, 1, 10 mg/mL) were applied daily, as indicated. Dexamethasone at 0.12% was a positive control. Re-challenge of 7% DNCB was performed on day 6. Mice were sacrificed on day 11. Vehicle (vaseline) only treatment was adopted as blank. **(B)** Scratching duration and **(C)** severity of dermatitis were assessed daily. Values are expressed in mean ± SEM, *n* = 6. The quantifications of pro-inflammatory cytokines in collected skin: **(D)** TNF-α, **(E)** IL-6 and **(F)** IL-1β, and **(G)** skin barrier filaggrin protein (FLG) are shown. Dose dependence of NANA (1, 10, 100 mM), EBN extract and digest (0.1, 1, 10 mg/mL) were determined, as indicated. Dexamethasone at 0.12% was a positive control. Values are expressed as the fold change to normalized basal activity set at 1, or percentage of change to normalized basal activity set at 0%, in mean ± SEM, *n* = 6. Statistically significant results are marked with *p < 0.05, **p < 0.01 and ***p < 0.001 against the DNCB-treated group.

To validate the anti-inflammatory function of EBN in skin, the DNCB-induced dermatitis in mouse model was employed. The treatment procedure was shown in Figure 7A. The severity of dermatitis was evaluated by a simple scoring system with minor modification from SCORAD (Nantes and Bordeaux, 1993). In addition to the six intensity criteria, as listed in SCORAD, e.g., erythema, edema/papulation, oozing/crusts, excoriations, lichenification, and dryness, two additional criteria were included, i.e., vesicle and pigmentation/depigmentation. The score was given daily for existence (0: absence, 1: existence), rather than to measure the extent of intensity, as to avoid discrepancy. Besides, the number and duration (how long the scratch lasts) of scratching were counted for 30 min daily in the whole timeline of experiment. The scratch duration in DNCB-sensitized group was significantly increased by 2 folds, from ∼5 s in control to ∼10 s under DNCB (Figure 7B). Dexamethasone, serving as a control, suppressed the scratching time. The topical applications of EBN, including NANA, extract and digest, reduced the duration, significantly (Figure 7B). NANA showed the best in reducing the itchiness. In addition, the severity score was increased by over 25 folds after DNCB treatment (Figure 7C). The applications of NANA, EBN extract and digest could markedly reduce the severity scores. The score was greatly reduced by ∼80% at high dose of EBN application, similar to that of dexamethasone (Figure 7C).

The amounts of pro-inflammatory cytokines, TNF-α, IL-1β and IL-6, were assessed by ELISA assays from the isolated skin of treated mice. As expected, these cytokines were robustly induced in DNCB-treated mice (Figures 7D–F). The treatments of EBN preparations, in all scenarios, suppressed the expressions of TNF-α, IL-1β and IL-6 in the skin: the significant suppression could be revealed by EBN digest (Figures 7D–F). On the other hand, the amount of filaggrin was decreased by ∼30% by the treatment of DNCB; however, the reduction could be markedly reversed by the EBN preparations (Figure 7G).

**FIGURE 8:**
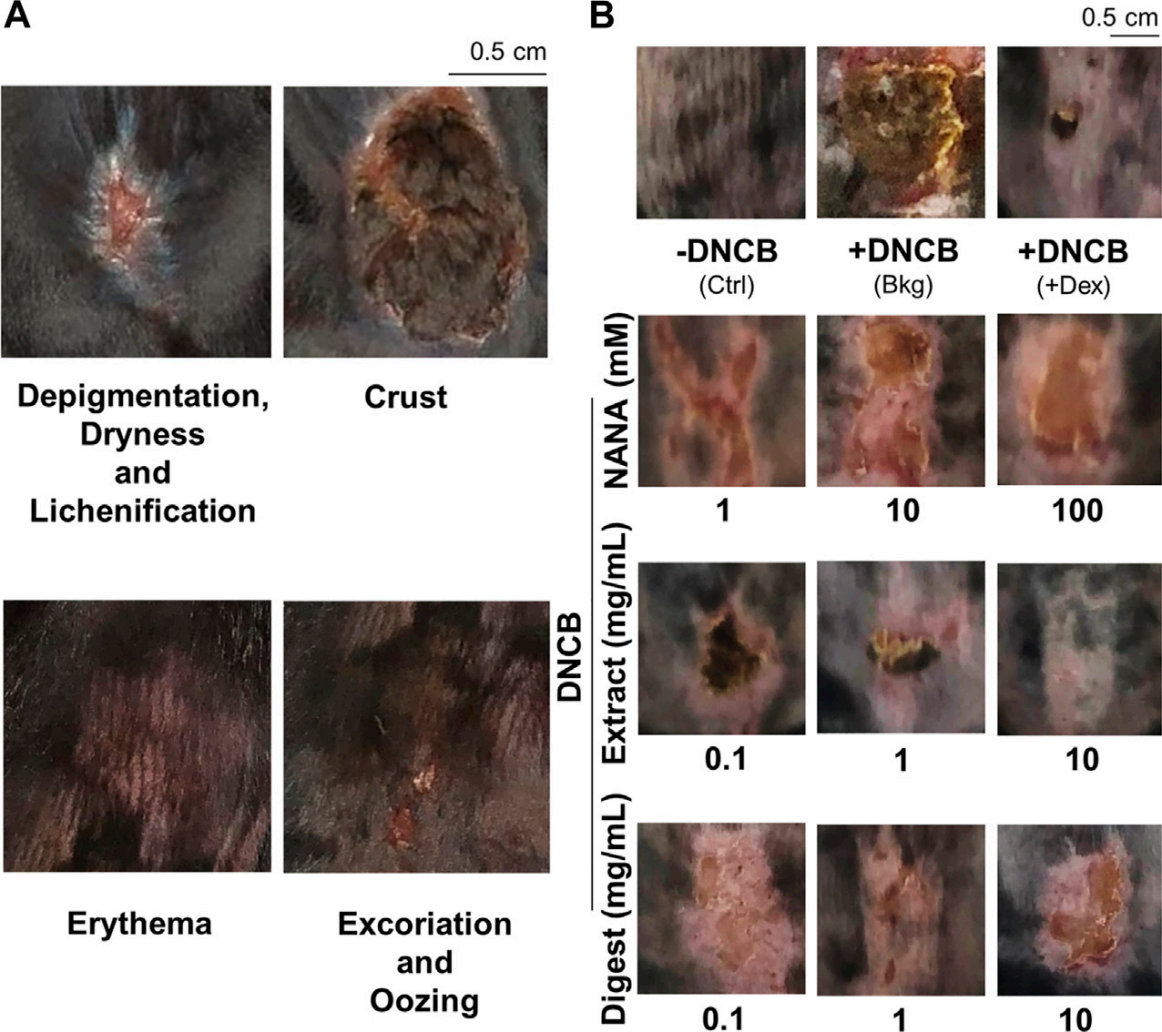
EBN lowers intensity of symptoms in DNCB-induced atopic dermatitis mice. The treatment of mice skin was followed as in Figure 7A. **(A)** Representative images of skin features in DNCB-induced atopic dermatitis mice are shown. **(B)** Representative images of mice under different treatments at day 11 are shown.

**FIGURE 9:**
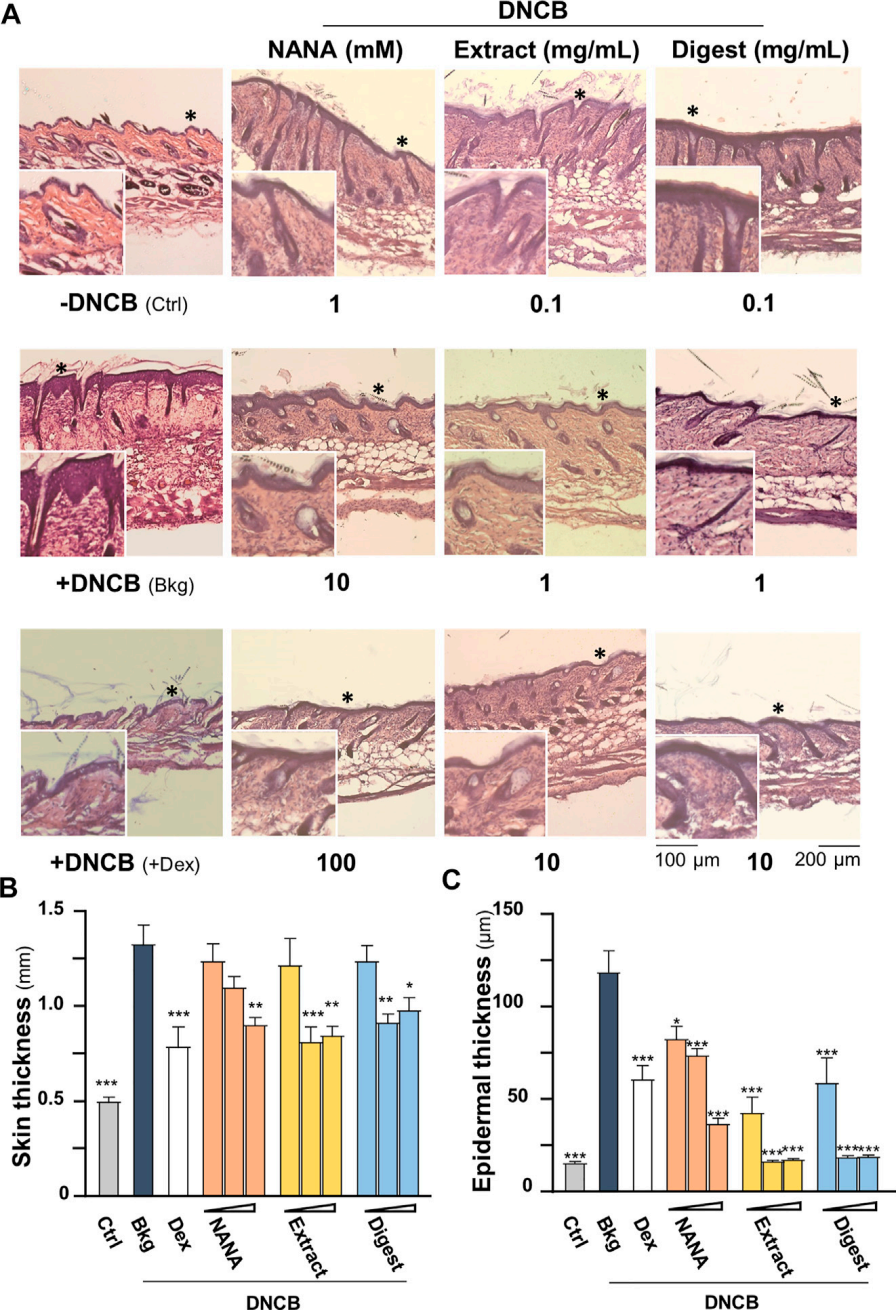
EBN alleviates skin thicknesses in DNCB-induced atopic dermatitis mice. The treatment of mice skin was followed as in Figure 7A. **(A)** Representative H&E stain images of mouse skin section under different treatments are shown, * marks for magnification. The thicknesses of **(B)** skin and **(C)** epidermal layer under different treatments were measured at five randomly selected area in the 10X field images. Dose dependence of NANA (1, 10, 100 mM), EBN extract and digest (0.1, 1, 10 mg/mL) were determined, as indicated. Dexamethasone at 0.12% was a positive control. Values are expressed in mm or μm, in mean ± SEM, *n* = 6. Statistically significant results are marked with *p < 0.05, **p < 0.01 and ***p < 0.001 against the DNCB-treated group.

The dorsal skin of DNCB-sensitized mice was recognized by excoriations, dryness, crust, erythema, oozing and depigmentation (Figure 8A). The overall severity, determined by summarizing daily scores after inspection of skin features throughout the time course, were significantly ameliorated after treatments of NANA and EBN preparation, respectively (Figures 7C, 8B). In sacrificed mice, the skin showed an increase of its thickness almost by 3 folds under the influence of DNCB (Figures 9A,B). The EBN preparations, similar to dexamethasone, were able to reduce markedly the skin thickness (Figures 9A,B). In H&E staining of skin epidermal layer, the epidermis displayed strong purple color, as shown in Figure 9A. The quantitation showed that the epidermal thickness significantly increased by over 6 folds under the exposure to DNCB (Figure 9C). The increased epidermal thickness was reduced by treatments of NANA, EBN extract, and EBN digest in a dose-dependent manner. Again, the digest showed the best effectiveness (Figure 9C).

**FIGURE 10:**
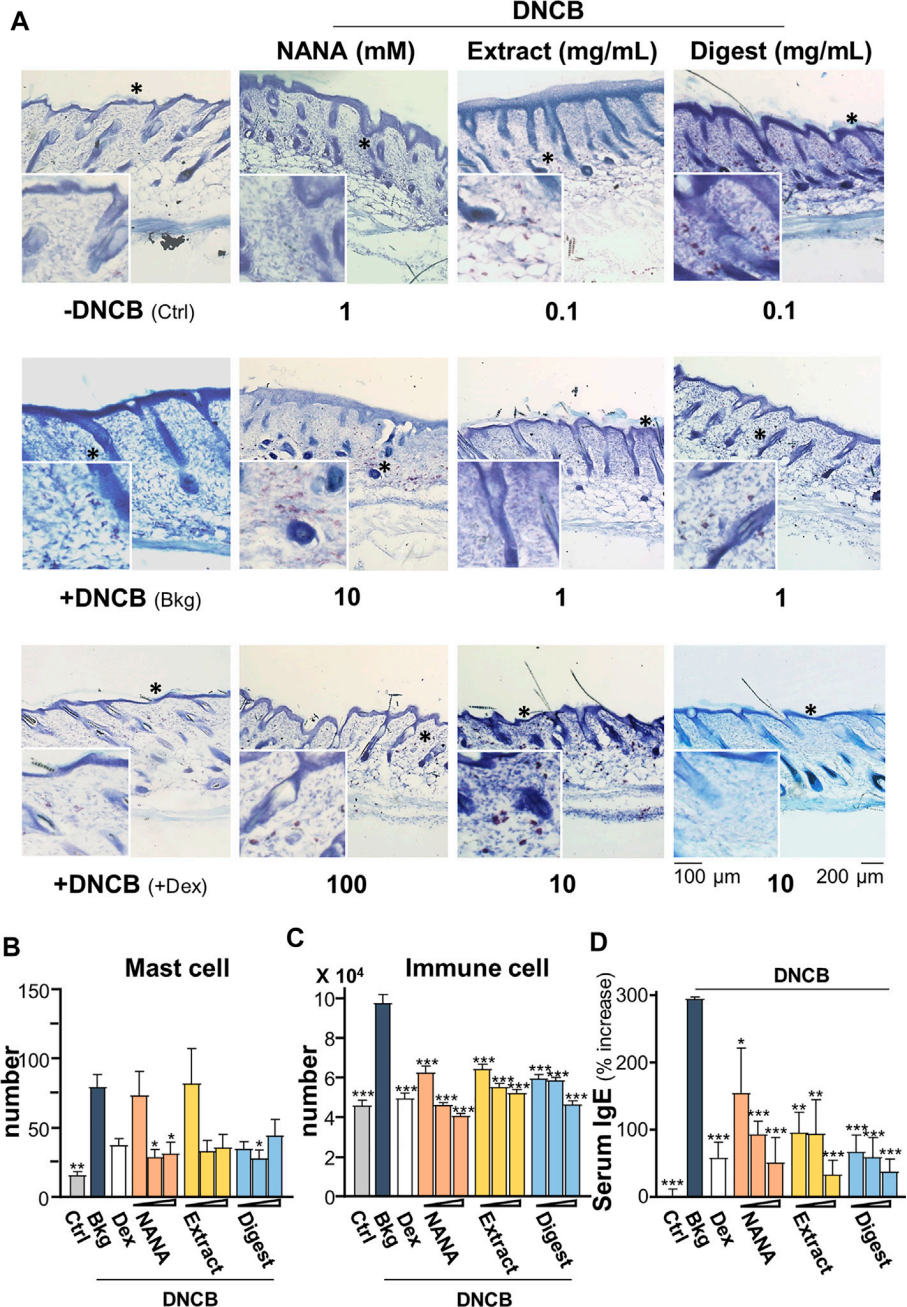
EBN reduces number of immune cells in DNCB-induced atopic dermatitis mice. The treatment of mice skin was followed as in Figure 7A. **(A)** Representative toluidine blue stain images of mouse skin section under different treatments are shown, * marks for magnification. The numbers of **(B)** mast cell and **(C)** immune cells under different treatments were counted per mm^2^. **(D)** The quantification of serum IgE in collected blood is shown. Dexamethasone at 0.12% was a positive control. Values are expressed in mean ± SEM, *n* = 6. Statistically significant results are marked with *p < 0.05, **p < 0.01 and ***p < 0.001 against the DNCB-treated group.

In addition, the mast cells in skin were stained by toluidine blue to purplish red color (Figure 10A); while immune cells were stained as strong bluish-purple color under H&E stains (Figure 9A). The challenge of DNCB sensitizer increased the number of mast cell significantly by over 4 folds (Figure 10B), as well as the total number of immune cells by over 2 folds (Figure 10C). All EBN treatments showed significant suppression to the number of mast cell or immune cell, showing at almost 60% reduction at the highest dosage application. The level of serum IgE was reported to regulate the expression of FcεRI receptor on mast cells and basophils (Rosenwasser, 2011; Greer et al., 2014). The challenge of DNCB induced the serum IgE to ∼300%, and the treatments of NANA and EBN extract/digest significantly suppressed the level of serum IgE (Figure 10D).

## Discussion

The consumption of EBN has been widely adopted as health food supplement for improving skin property. Several lines of evidence support this specific application of consuming EBN. The cell proliferating activity of EBN has been reported (Kong et al., 1987). Besides, EBN extract stimulates the wound healing in cultured fibroblasts and keratinocytes (Hwang et al., 2020). However, the identity of protein/peptide responsible for this activity has never been revealed so far in EBN (Wong et al., 2018b; Li et al., 2021). NANA, a major form of sialic acid in EBN, has been shown to reduce melanin production in skin: the reduction of melanin was mediated by inhibition of tyrosinase, an enzyme to synthesize melanin (Chan et al., 2015). Furthermore, EBN could reduce water loss, wrinkle area and dermal thickness of skin (Terazawa and Shimoda, 2020). The skin moisturizing effect, exerted by EBN, was markedly enhanced by enzymatic digestion of EBN extract: this moisturizing effect was proposed to be mediated by increased expressions of filaggrin and filaggrin-2 in keratinocytes (Lai et al., 2021). In addition, the skin beneficial function of EBN in fighting against inflammation is being illustrated here in both *in vitro* and *in vivo* models. The EBN-mediated anti-inflammatory responses in keratinocytes could be triggered by different signals: 1) inducing expression of filaggrin and filaggrin-2; 2) suppressing production of pro-inflammatory cytokines via a reduction of NF-κB signaling; 3) suppressing phosphorylations of p38 MAPK and JNK; and 4) reducing the production of ROS.

Skin epidermis composes of keratinocytes, Langerhans cells, melanocytes, and different cell types in responding to external stimuli for the maintenance of skin homeostasis (Ogawa, et al., 2018). The pro-inflammatory cytokine TNF-α can induce inflammatory signaling in cultured keratinocytes, as to mimic AD (Choi & Hwang, 2019). TNF-α induces the transcription of inflammation-related genes via activation of NF-κB pathway, in which the *trans* activation of NF-κB is involving the signaling of p38 and JNK (Pastore et al., 2005). Additionally, TNF-α promotes a rapid metalloproteinase-mediated TGF-α shedding from keratinocyte membrane, attributed to EGFR trans-activation and accelerated epithelial cell regeneration (epidermal hyperplasia), which aggravates skin inflammatory conditions (Mascia et al., 2003). As shown here, NANA and digested product of EBN significantly inhibited the expressions of pro-inflammatory cytokines, as well as the phosphorylations of Iκ-Bα, NF-κB, p38 and JNK. Besides, NANA and EBN preparation did not inhibit the TNF-α driven EGFR activation, which might thus prevent from dampening the inflammatory responses. Besides, a high level of TNF-α-induced ROS was proposed in switching the NF-κB-mediated cell survival into cell death by activating JNK-mediated signaling (Blaser, et al., 2016), and subsequently the translocation and activation of HIF-α (Van Uden et al., 2011). The current results showed that EBN digest significantly suppressed intracellular level of ROS, as well as promoting the transcription of Nrf2-ARE signaling pathway. Further evidence in inhibiting the transcription of TNF-α-induced HIF-α signaling validated the anti-inflammatory functions of NANA and EBN preparation. Additionally, NANA and EBN digest showed robust efficiency in increasing expressions of skin barrier proteins, filaggrin and filaggrin-2: these two proteins were markedly reduced by TNF-α via JNK signaling (Kim et al., 2011). Thus, NANA and EBN preparation could rescue the skin barrier proteins by acting on PPARγ, an upstream transcription factor of filaggrin and filaggrin-2 (Ye, 2008).

An elevated level of IgE was found in patients suffering from AD (Stone, et al., 2010), and which was correlated to the degree of dermatitis (Laske & Niggemann, 2004). Several immune cells possess high- (FcεRI) and low-affinity (FcεRII) receptors of IgE. FcεRI is expressed on mast cell and basophil, whereas FcεRII is expressed in eosinophil. During the immune sensitization, mast cell and basophil are activated via high-affinity FcεRI receptor, and thereafter which facilitates the degranulation of mast cell and basophil, as to induce the release of histamine. Besides, TNF-α is also being released from mast cell and eosinophil, facilitating the cell infiltration to infected sites. Here, the topical applications of EBN on AD mouse skin could significantly suppress: 1) the number of immune cells, e.g., Langerhans cell, basophil, eosinophil and mast cell; 2) the production of pro-inflammatory cytokines; 3) the serum level of IgE; and 4) the activation of canonical NF-κB signaling pathway.

Two active ingredients in EBN, digested peptide and NANA, could be accounted possibility for the aforementioned activities. Chemical analysis has shown that the enzymatic digested EBN was enriched with small peptide and free NANA (Wong et al., 2018a). NANA is a major form of sialic acid in EBN, accounting ∼10% in dried weight (Kathan & Weeks, 1969; Wong, et al., 2018b). As proposed by Varki (2008), sialic acid is presenting as ligand for selectins, which mediates the infiltration of lymphocytes and recruitment of immune cells in skin during inflammation. Moreover, sialic acid is also a ligand for sialic-acid-binding-immunoglobulin-like lectins (Siglec), a family of cell adhesion molecules responsible for immune response. In line to this notion, Siglec-8, an I-type lectin expressing only in eosinophil, basophil and mast cell, has recently been reported for its potency as a therapeutic target for AD (Bochner, 2016). Besides, the contents of NANA in EBN extract and digest are at ∼4.32% and ∼11.43% (w/w), as determined by LC-MS/MS, respectively, which are corresponding to ∼0.014 mM and ∼0.037 mM free NANA in 100 μg/mL EBN extract and digest. This small amount of free NANA in the tested EBN extract/digest however could not account for the full EBN functions, as reported here. Thus, the small functional peptides, released from the enzymatic digestion of EBN, should account for the anti-inflammatory responses.

Our current study has apparently evidenced the potency of EBN digested peptides in developing into anti-inflammatory skincare products. However, we have not identified the key anti-inflammatory player composed in EBN digest. We believe the pepsin digestion has completely hydrolyzed the linked glycan in EBN protein, leaving alone the functional peptide(s) that exerts the anti-inflammatory response in skin. In future study, we aim at identifying the anti-inflammatory peptide(s), as well as unraveling the chemical modifications behind pepsin digestion leading to release of potent peptide(s).

In treating AD, topical steroid is widely adopted as the common therapeutic treatment. However, adverse side effects are frequently reported of usage of steroid, such as epidermal thinning, reduced potency in long-term usage, and drug dependence (Coondoo et al., 2014). Although other treatments of AD have been employed, there is still a surging demand in looking for natural products, or its active compounds, as substitutes for the skin treatment. A recent FDA-approved drug Dupilumab, an antibody targeting to inhibit IL-4 and IL-13, has been adopted to treat severe AD patients; however, nearly 20% subjects experienced adverse events during phase III clinical trials of Dupilumab (Gooderham et al., 2018). On the other hand, the price of Dupilumab is high, over US$ 1,000 per injection is required once in every 2 weeks. In contrast, the EBN-derived products possess the advantages of lower toxicity and hypo-allergenicity, and more importantly, it should be more affordable by most patients. Besides, the topical application of low molecular weight peptides should possess higher bioavailability, deeper skin penetration, lower toxicity, degradation and hypo-allergenicity (Aguilar-Toalá, et al., 2019). Our results support a great potential of EBN in developing into anti-inflammatory and moisturizing skincare products in treating AD, a medical problem does not have effective cure today.

## Data Availability

The raw data supporting the conclusion of this article will be made available by the authors, without undue reservation.

## Supplementary material

SUPPLEMENTARY FIGURE S1 Chemical standardization of EBN extract and digest. **(A)** Representative MS spectrum of NANA measured in EBN extract is shown. **(B)** Multiple reaction monitoring (MRM) spectrum of NANA obtained from EBN extract. **(C)** HPLC chromatogram obtained from gel filtration chromatography of EBN extract (10 mg/mL, injection volume = 10 μL) and digest (10 mg/mL, injection volume = 100 μL) under UV wavelength of 214 nm are shown. The elution profile of a protein marker mix containing α-melanocyte-stimulating hormone (1.7 kDa), aprotonin (6.5 kDa), ribonuclease A (13.7 kDa), carbonic anhydrase (29 kDa), ovalbumin (43 kDa), albumin (75 kDa) and blue dextran (>2,000 kDa) are marked. **(D)** HPLC chromatogram obtained from reverse phase chromatography of EBN digest (10 mg/mL, injection volume = 10 μL) under UV wavelength of 214 nm are shown. Peaks labelled with retention time served as authentication markers, as described (Wong et al., 2017). (E) Cultured HaCaT cells were treated with NANA (0.01, 0.05, 0.1, 0.5, 1, 5 mM), EBN extract or digest (1, 5, 10, 50, 100, 500 μg/mL) respectively. Cell viability test was performed by MTT assay. Values were in mean ± SEM, *n* = 6.

SUPPLEMENTARY FIGURE S2 NANA induces gene expressions responsible for skin moisturization. The mRNA levels of filaggrin, filaggrin-2 and PPARγ in cultured keratinocytes after 24-h treatment of NANA are shown. NANA (0.05, 0.1, 1 mM) were applied, as indicated. CaCl_2_ at 0.16 mM, and linoleic acid (C18:2, 30 μM) were positive controls, as indicated. All values are in mean ± SEM, *n* = 6. Statistically significant results are marked with * *p* < 0.05, ** *p* < 0.01 and *** *p* < 0.001 against the control.
