# Supplementary figures and images for: Edible bird’s nest, an Asian health food supplement, possesses anti-inflammatory responses in restoring the symptoms of atopic dermatitis: An analysis of signaling cascades
Source: Front Pharmacol. 2022 Sep 20;13:941413. doi: 10.3389/fphar.2022.941413 (PMC9531170; doi:10.3389/fphar.2022.941413)

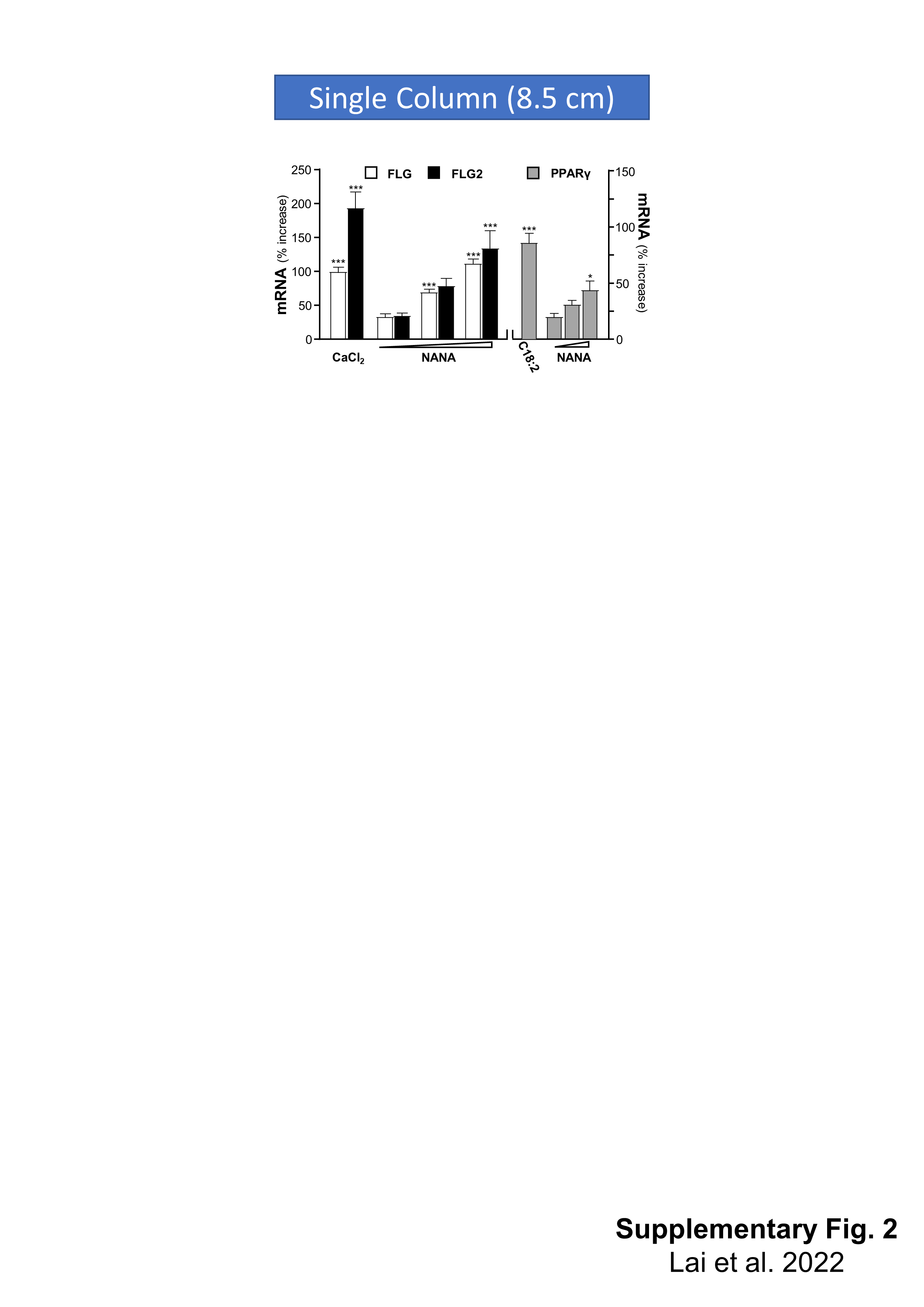

Supplement: Supplementary file 1 [file Image2.tif]

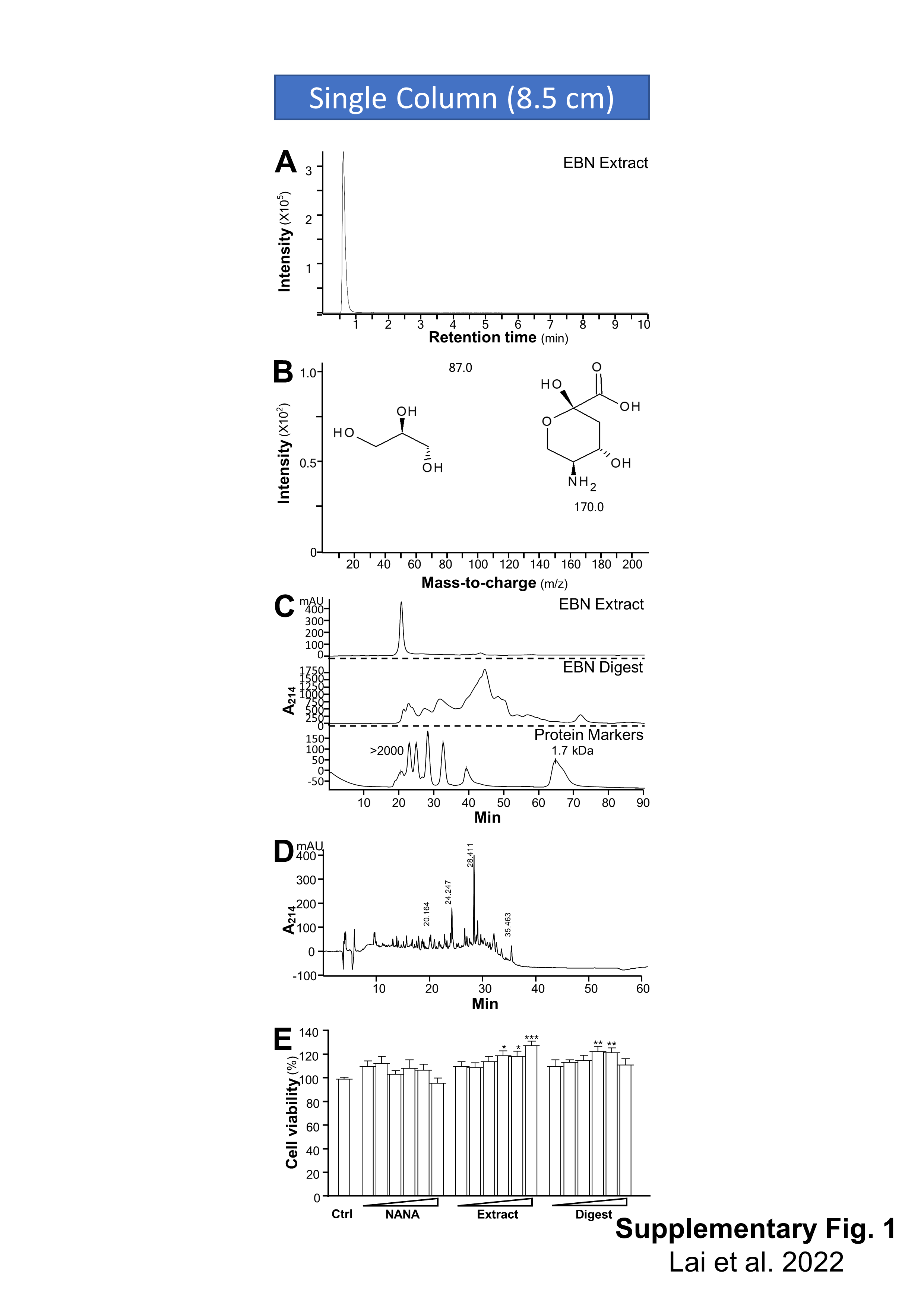

Supplement: Supplementary file 2 [file Image1.TIF]
